# Short Chain Fatty Acids: Essential Weapons of Traditional Medicine in Treating Inflammatory Bowel Disease

**DOI:** 10.3390/molecules29020379

**Published:** 2024-01-12

**Authors:** Yuan Yao, Yongchao Liu, Qiuyun Xu, Liming Mao

**Affiliations:** 1 Department of Immunology, School of Medicine, Nantong University, 19 Qixiu Road, Nantong 226001, China; 2231310020@stmail.ntu.edu.cn (Y.Y.); 2231310015@stmail.ntu.edu.cn (Y.L.); 2 Basic Medical Research Center, School of Medicine, Nantong University, Nantong 226019, China

**Keywords:** ulcerative colitis (UC), short-chain fatty acids, traditional drugs, mechanism of action, natural compounds

## Abstract

Inflammatory bowel disease (IBD) is a chronic and recurrent intestinal inflammatory disease, mainly including Crohn’s disease (CD) and ulcerative colitis (UC). In recent years, the incidence and prevalence of IBD have been on the rise worldwide and have become a significant concern of health and a huge economic burden on patients. The occurrence and development of IBD involve a variety of pathogenic factors. The changes in short-chain fatty acids (SCFAs) are considered to be an important pathogenic mechanism of this disease. SCFAs are important metabolites in the intestinal microbial environment, which are closely involved in regulating immune, anti-tumor, and anti-inflammatory activities. Changes in metabolite levels can reflect the homeostasis of the intestinal microflora. Recent studies have shown that SCFAs provide energy for host cells and intestinal microflora, shape the intestinal environment, and regulate the immune system, thereby regulating intestinal physiology. SCFAs can effectively reduce the incidence of enteritis, cardiovascular disease, colon cancer, obesity, and diabetes, and also play an important role in maintaining the balance of energy metabolism (mainly glucose metabolism) and improving insulin tolerance. In recent years, many studies have shown that numerous decoctions and natural compounds of traditional Chinese medicine have shown promising therapeutic activities in multiple animal models of colitis and thus attracted increasing attention from scientists in the study of IBD treatment. Some of these traditional Chinese medicines or compounds can effectively alleviate colonic inflammation and clinical symptoms by regulating the generation of SCFAs. This study reviews the effects of various traditional Chinese medicines or bioactive substances on the production of SCFAs and their potential impacts on the severity of colonic inflammation. On this basis, we discussed the mechanism of SCFAs in regulating IBD-associated inflammation, as well as the related regulatory factors and signaling pathways. In addition, we provide our understanding of the limitations of current research and the prospects for future studies on the development of new IBD therapies by targeting SCFAs. This review may widen our understanding of the effect of traditional medicine from the view of SCFAs and their role in alleviating IBD animal models, thus contributing to the studies of IBD researchers.

## 1. Introduction

Inflammatory bowel disease (IBD), mainly including Crohn’s disease (CD) and ulcerative colitis (UC), is a non-specific chronic gastrointestinal inflammatory disease with unclear etiology [1]. IBD is characterized by recurrent symptoms, including mucopurulent bloody stools, weight loss, abdominal spasm, fatigue, anemia, extraintestinal symptoms, and multiple complications such as joint pain and arthritis. At the same time, the body produces large amounts of cytokines, proteolytic enzymes, and free radicals, which eventually lead to inflammation and ulcers [1,2]. According to a study in the United States, IBD is listed as the fifth most expensive gastrointestinal disease, and its patients accumulate additional out-of-pocket costs of nearly $500 million per year in treatment [3]. The treatment of this disease is not ideal due to the lack of medicine with enough efficiency and efficacy, although recent studies have made significant progress in understanding the pathogenesis of IBD. Therefore, it is an urgent and challenging task to study the pathophysiological mechanisms of IBD under the complex interaction of various factors such as environmental changes, immune disorders, and intestinal flora [1].

The gut microbiota and their diverse metabolites have close interactions with the host and impact the susceptibility of the host to many diseases. The symbiotic gut bacteria produce a variety of metabolites, including short-chain fatty acids (SCFAs), tryptophan catabolites, essential vitamins such as B and K vitamins, phenolic acids, and bile acids. Among these metabolites, SCFAs are arousing increasing concerns of IBD researchers since the observations of various beneficial effects of these metabolites on multiple intestinal conditions and becoming the most well-studied microbial metabolites associated with IBD [4,5]. SCFAs usually contain less than six carbon atoms, mainly including formic acid, acetic acid, propionic acid, butyric acid, and valeric acid. They are the secondary metabolites produced by the fermentation of intestinal dietary fiber, such as peptides, proteins, resistant starch, and undigested fiber. This type of metabolite is an important part of the fecal samples of both healthy and diseased conditions. The production of SCFAs is regulated and affected by many factors, such as host nutrition, the presence/absence of specific symbiotic bacteria, and transgenic diversity in concentration [4]. Among the three major types of SCFAs, propionate and acetate are mainly produced by Bacteroidetes, while the production of butyrate is mainly mediated by Firmicutes [6]. Propionic acid can be produced through the lactic acid pathway of Firmicutes or the succinic acid pathway of Bacteroidetes [7].

The SCFAs play an important regulatory role in intestinal cell function and may also be associated with multiple intestinal pathophysiological processes such as inflammatory responses. The compositions, relative ratios, and activities of these SCFAs may be variable in different parts of the intestine and thus have various impacts on the physiological conditions of the intestine [8]. First of all, SCFAs contribute to nutritional balance and cellular integrity of the intestine by providing a molecular source for phospholipid synthesis [9]. In eukaryotic hosts, SCFAs can be used as energy sources by colon cells and can also be transported to blood circulation and other tissues and therefore act as important promoters and fuels of intestinal epithelial cells, which strengthen intestinal barrier function and prevent intestinal inflammation [10,11]. Furthermore, some of the SCFAs play a role in maintaining the structure of gut microbiota and the integrity of intestinal epithelium and may thus aid the function of the intestinal epithelial barrier. Studies have shown that SCFAs can reduce the pH value of the intestine while inhibiting the growth of destructive bacteria [12]. Of note, some studies have revealed that the ecological imbalance of IBD patients is related to the impaired SCFA fermentation pathway. By comparing the affected samples with healthy individuals, the researchers found that the bacteria that ferment fibers and produce SCFAs in the mucosa and feces of IBD patients usually showed a decrease [8]. More and more evidence suggests that gut microbiota disruption affects the key physiology of the host from metabolism to immune response, and the production of SCFAs is closely related to the risk of IBD [11].

According to some studies in recent years, many compounds and decoctions in traditional medicine show antioxidant and anti-inflammatory activities, thus exhibiting potential anti-IBD effects. Some of the medicines affect the production of SCFAs and thereby suppress inflammatory conditions in the colon by affecting multiple biological processes associated with gut homeostasis. Therefore, regulating SCFA content and composition might be an important approach to IBD treatment. Here, we first provide an overview of the major functions of SCFAs in colonic inflammation. On this basis, we summarize the effects of a variety of traditional medicines on the production of SCFAs. Meanwhile, we will discuss the potential mechanisms of action, the related regulatory factors, and signaling pathways employed by SCFAs to achieve their functions. In addition, we provide our understanding of the possible limitations of current research and propose prospects for future studies in evaluating the potential of SCFAs in developing new anti-IBD drugs.

## 2. SCFA-Mediated Immune Regulation Plays a Key Role in Maintaining Intestinal Homeostasis

**Figure 1:**
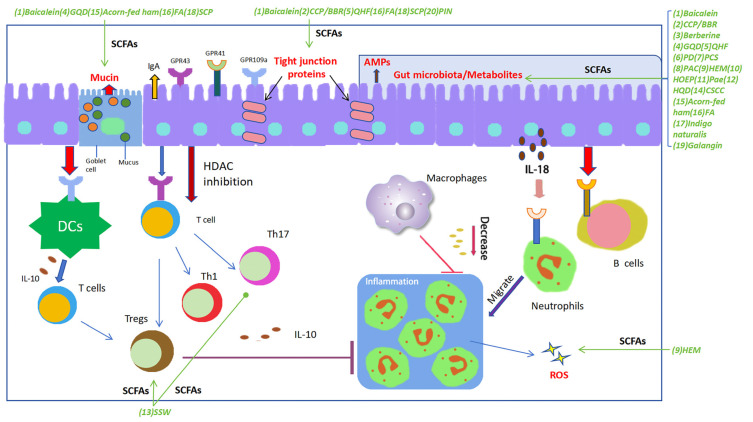
SCFAs play a pivotal regulatory role in maintaining gut homeostasis. SCFAs potentially play an essential role in each stage of the inflammatory process and tissue healing, exerting regulatory effects on the functionality of nearly all types of immune cells, thereby demonstrating their immunomodulatory impact. SCFAs contribute to maintaining the integrity of the intestinal barrier by promoting the proliferation of various epithelial cells, upregulating the expression of tight junctions in intestinal epithelial cells, and facilitating the secretion of barrier-supporting proteins such as IgA, AMPs, and mucins, meanwhile regulating the composition and structure of gut microbiota and oxidative stress. Furthermore, SCFAs exhibit various immunomodulatory actions: modulating the differentiation and function of Th17, Th1, and Tregs; inhibiting intestinal macrophages from producing pro-inflammatory cytokines by suppressing histone deacetylase (HDAC); inducing chemotaxis of neutrophils to the inflammatory site and enhancing their phagocytic activity; and stimulating intestinal B cells to produce IgA. The decoctions/compounds were marked in green. Green arrows were used to indicate the effects of a decoction or a compound on the targets. Abbreviations: (1) Baicalein; (2) Polysaccharides (CCP) and berberine (BBR); (3) Berberine; (4) Gegen Qinlian Decoction (GQD); (5) Qingchang Huashi Formula (QHF); (6) Pulsatilla decoction (PD); (7) Pulsatilla chinensis saponin (PCS); (8) Polysaccharides from Astragalus membranaceus and Codonopsis pilosula (PAC); (9) Polysaccharide of Hericium erinaceus mycelium (HEM); (10) Herba Origani Extract Pulvis (HOEP); (11) Paeonol (Pae); (12) Huangqin Decoction (HQD); (13) Sishen Wan (SSW); (14) Composite Sophora colon-soluble Capsule (CSCC); (15) Acorn-fed ham; (16) Fermented astragalus (FA); (17) Indigo naturalis; (18) Schisandra chinensis polysaccharide (SCP); (19) Galangin; (20) Pinocembrin (PIN).

Some SCFAs, especially acetate, propionate, and butyrate, have been shown to play a pivotal role in preventing intestinal inflammation via various mechanisms [13]. Here, we describe these advances mainly through three aspects. First of all, SCFAs may affect epithelial proliferation and differentiation to change the integrity and barrier function of the intestinal epithelium. Furthermore, the generated SCFAs in the gut can stimulate the epithelial cells to produce a variety of gut-protective molecules such as secretive IgA, mucin, and antimicrobial peptides (AMPs), to protect the epithelium against pathogenic microorganisms and virulent substances in the gut. Additionally, the immune regulatory role of some SCFAs may induce alterations of gut immune responses via various signaling pathways. All these effects of SCFAs may contribute to the quiescence of the gut inflammation and support an establishment of homeostasis of the gut. We will discuss the recent advances of SCFAs regarding multiple functions as follows (Figure 1).

### 2.1. Regulates the Functions of the Intestinal Epithelial Barrier

SCFAs have a role in promoting the healing of the intestinal epithelial barrier, mainly composed of cylindrical epithelial cells and other functional cell types such as goblet cells. Previous studies have shown that some of the SCFAs affect the functions of multiple IECs via various mechanisms to regulate the barrier function of epithelium [14]. For instance, Singh et al. showed that butyrate could promote intestinal epithelial cells to produce IL-18 [15], a cytokine that can enforce the barrier function of epithelium by promoting cell proliferation [16]. The researchers also showed that the effect of butyrate was mediated by G protein-coupled receptor GPR109a, a receptor for butyrate in the colon. Deficiency of GPR109a could induce an increase of colonic inflammation in mouse model and administration of the agonist of GPR109a, Niacin, could ameliorate colonic inflammation in a GPR109a dependent manner, further strengthened the effect of butyrate–GPR109a axis in regulating epithelial barrier function. Moreover, a study by Deleu et al. [17] evaluated the impact of acetate on intestinal barrier integrity, an SCFA that was considered to be less toxic to epithelial cells. Using organoid-based monolayer cultures obtained from UC patients, the researchers found that a high concentration of acetate could induce proliferation of epithelial cells indicated by enhanced level of cell proliferation marker, MKI67, in the cells. Meanwhile, acetate treatment also significantly enhanced the production of barrier genes such as MUC2 and CLDN1. Additionally, in a study by Peng et al., the researchers employed a cellular model of intestinal barrier established by Caco-2 cell monolayer and disclosed that butyrate could induce an increase of transepithelial electrical resistance and a decrease of inulin permeability, indicating an effect of butyrate in up-regulating intestinal barrier function [18].

Of note, the intercellular tight junction (TJ) proteins play an essential role in the integrity of the barrier [19] and act as a major component of the intestinal mucosal mechanical barrier [6]. These proteins distribute between adjacent intestinal epithelial cells and thus play a role in preventing harmful substances from entering the submucosa, which plays an extremely important role in maintaining intestinal health [20]. The major components of the TJ are transmembrane proteins, including occludin, claudins, junctional adhesion molecules (JAM), and auxiliary cytoplasmic proteins such as occlusive bands (ZOs) [21,22]. Studies have shown that SCFAs can maintain epithelial integrity and restore normal barrier function. SCFAs regulate the permeability between intestinal cells by regulating the expression of tight junction proteins. For instance, the study by Saleri et al. [23]. showed that different SCFAs regulated the production of specific TJ proteins. Using porcine intestinal epithelial cells, the researchers disclosed that butyrate could selectively up-regulate the production of ZO-1 and occludin, while it had minimal role on the level of claudin 4. Acetate significantly enhanced the levels of occludin, and claudin 4, but had no effect on ZO-1. In comparison, lactate could only affect the level of ZO-1. Only propionate could promote the production of the three TJ proteins. Some studies provided further evidence that SCFAs may affect the assembly of TJ proteins. As shown in Miao et al.’s study [24], sodium butyrate promoted the reassembly of TJ in Caco-2 monolayers via inhibiting MLCK/MLC2 pathway and phosphorylation of PKCβ2.

### 2.2. Regulates Barrier-Supporting Proteins

Besides the role of SCFAs in regulating epithelial barrier-forming genes, these molecules have a role in promoting the expression of multiple barrier-supporting proteins, including the mucus and some defense proteins such as immunoglobulin (Ig). In a study by Deleu et al., the researcher used a organoid-based epithelial monolayer culture to evaluate the potential role of acetate on the production of barrier-supporting proteins and showed that stimulation with acetate could enhance the production of MUC2 by the cells, demonstrating the impact of acetate on mucin production and barrier protection [17]. Another in vitro study using human cell models by Willemsen et al. revealed that butyrate and propionate could also stimulate the production of MUC2 by epithelial goblet cells and this process was mediated by prostaglandin E [25]. Although the exact signaling pathways employed by SCFAs in promoting mucin production have not been clearly elucidated, an early study by Poul et al. [26] pointed out that several SCFAs, including acetate, propionate, and butyrate, could act as ligands of G protein coupled receptors such as GPR41 and GPR43. The exact contribution of both receptors and their downstream signaling pathways in mucin production requires further clarification. Intestinal IgA plays an essential role in regulating gut homeostasis by binding and facilitating the removal of pathogenic bacteria and microbial factors and meanwhile restoring the commensal bacteria [27]. The study by Wu et al. [28] disclosed that acetate derived from microbiota can trigger IgA production in the intestine and the process is mediated by the receptor GPR43 [29]. Another study by Takeuchi et al. [30] disclosed that acetate not only enhances IgA production in the intestine but also modulates the binding specificity of IgA to certain commensal microorganisms such as Enterobacterales. However, if other common receptors of SCFAs such as GPR41 and GPR109a play a role in acetate-mediated IgA production and the anti-microbial activity of IgA need to be further determined. Moreover, the SCFAs may have a role in regulating IgA secretion in the saliva. In this line of evidence, Yamamoto et al. [31] found that the ingestion of polydextrose can promote SCFA absorption and thus lead to enhanced salivary IgA levels in rats. To date, no direct evidence demonstrates the effects of propionate and butyrate on IgA production, while, since propionate also binds to GPR43, it may induce the production of IgA, but the role needs to be further validated in future studies. Some other studies suggest that the impact of SCFAs on IgA production may differ in various tissues or different inflammatory conditions. An example of this notion is supported by the study of Chai et al. [32], which shows that reduced SCFA levels correlate with IgA builds up in the kidney of IgA nephropathy. Similarly, in a study by Tominaga et al. [33], the researcher showed that in division colitis the levels of SCFAs are also negatively correlated with IgA in the feces. Thus, the effect of SCFAs on IgA production may need to be further verified in more studies of different inflammatory environments. All these observations provided further evidence that SCFAs play an essential role in regulating the expression of barrier-supporting genes.

### 2.3. Regulates Gut Microbiota

The gut contains a large number of microbes with high complexity and heterogeneity, including bacteria, fungi, viruses, and other microbial populations [34]. The normal structure and composition of the microbiota may play an important role in maintaining intestinal homeostasis. Its dysregulation is associated with many human diseases, including IBD [9]. In normal conditions, the microorganisms in the gut may establish a symbiotic relationship with the host, and promote the fermentation of complex carbohydrates and the production of SCFAs to enhance the integrity of the intestinal barrier [35]. The SCFAs and SCFA-producing bacteria may in turn have a regulatory role in the composition and structure of the gut microbiota. For example, Wang et al. found that the loss of butyrate-producing *Faecalibacterium prausnitzii* was associated with the increased proportion of Bifidobacterium and the Lactobacillus in both fecal and biopsy specimens of IBD patients [36]. Moreover, the study of Kumari et al. [37] observed that the levels of butyrate-producing *Clostridium coccoides* and *Clostridium leptum* clusters were significantly reduced in fecal samples of UC patients. Additionally, SCFAs such as butyrate can induce the production of IL-18, which is involved in the synthesis of AMPs such as defensins, calprotectin, and lipocalin [38]. The AMPs play an essential role in suppressing the proliferation of some pathogenic bacteria such as staphylococcus. Both the composition of the microbiota and the levels of AMPs are important in the development of colonic inflammation [38]. Some of the AMPs are produced by epithelial cells during the inflammatory process and affect the progress of IBD [39]. Thus, gut microbiota and AMPs are important targets of SCFAs in regulating the development of IBD.

### 2.4. Regulates Immune Responses in the Gut

Precedent studies revealed the role of SCFAs in suppressing the inflammatory responses in the gut. It has been observed that some SCFAs can inhibit the recruitment of monocytes and macrophages, as well as neutrophils, by inhibiting the expression of chemokines and adhesion molecules, which can indicate its potential anti-inflammatory effect [40]. In a study using a mouse model, it was proved that propionate and butyrate can inhibit the maturation of DC, which is a bridge between the innate immune system and the adaptive immune system [41]. Furthermore, SCFAs can regulate the activity of mouse DCs, which can produce cytokines and interact with T cells. Under the stimulation of butyrate, DCs can inhibit the differentiation of IFN-γ-producing T cells [42]. Butyrate can also regulate the activity of mouse colon lamina propria macrophages, and inhibit the transcription of pro-inflammatory molecules such as Nos2, IL-6, and IL-12 [43], probably by suppressing the activation of NF-κB in the TLR ligand response [44]. Some other studies also support the role of butyrate in regulating the NF-κB pathway by inducing nuclear peroxisome proliferator-activated receptor (PPARγ) or by inhibiting histone deacetylase (HDAC) and proteasome activity [45,46].

Previous studies have demonstrated that the proportion of Tregs was increased in the intestine of IBD patients, especially in inflammatory lesions [47]. SCFAs can also play an immunomodulatory role in various T cells such as Th17, Th1, and Tregs in different cytokine environments [48]. As mentioned above, butyrate-mediated production of IL-18 can suppress Th17 differentiation in the gut and meanwhile enforce the function of Foxp3+ Treg cells [49]. This role of butyrate may be associated with its effect on regulating epigenetic modification, up-regulating histone H3 acetylation of Foxp3, and inducing Treg differentiation [50]. The HDAC inhibitory activity of butyrate also stimulates changes in gene expression in mouse DCs, including inhibition of IL-6 and IL-12, thereby affecting the polarization of Tregs [51].

Another function of SCFAs in immune regulation is manifested by its effect on regulating the production of immune regulatory molecules. A good example is its role in regulating the level of IL-22 in the intestine, an essential cytokine in regulating intestinal mucosal immunity. IL-22 may play pro-inflammatory and anti-inflammatory properties depending on the inflammatory microenvironment [52]. A study by Yang et al. reported that microbiota-derived SCFAs could promote the production of IL-22 by innate lymphoid cells (ILCs) and CD4+ T cells in the intestine [53].

As to the mechanisms of SCFAs in regulating immune responses, many studies have found the ability of SCFAs to bind to receptors such as GPR41, GPR43, and GPR109. The main GPCRs activated by SCFAs are GPR43 and GPR41, which lead to mitogen-activated protein kinase signaling and the production of chemokines and cytokines, and mediate protective immune response and tissue inflammation in mice [54]. SCFAs induce neutrophil chemotaxis and regulate phagocytosis and reactive oxygen species (ROS) production by activating GPR43 [54]. The production of IL-22 triggered by SCFAs was also mediated by the activation of GPR41 and inhibition of HDAC, which led to the up-regulation of aryl hydrocarbon receptor (AhR) and hypoxia-inducible factor 1α (HIF1α). The latter could bind to the Il-22 promoter and promote its transcription.

### 2.5. Regulates the Production of Reactive Oxygen Species (ROS)

ROS is a term to describe a series of oxygen-containing compounds with high oxidative properties produced during cell metabolism, mainly including hydroxyl radicals, superoxide anions, and hydrogen peroxide, which play a vital role in regulating many signaling pathways in maintaining the homeostasis of the intestine [55]. Some previous studies on the mechanism of SCFAs have focused on specific metabolites or genes involved in improving the therapeutic effect of colitis, such as reactive oxygen species (ROS) biosynthesis [56]. A previous study showed that SCFA induces apoptosis and activates autophagy in colitis [57]. This may be associated with the changes in ROS, which can be significantly induced in cells treated with high concentrations of SCFAs such as propionate [56]. Moreover, Maslowski et al. found that acetate can promote the release of ROS when added to mouse neutrophils by activating GPR43 [58]. The researchers believe that SCFAs may regulate inflammatory diseases by activating ROS to accelerate pathogen clearance [59]. In addition, SCFAs significantly altered the expression of genes involved in ROS production, such as PLIN5 [60], CDKN1A [61], UCP1 [61], COLIA1 [62], IMMP2L [63] and DUOXA2 [64]. These results suggest that SCFAs modulate the ROS signaling pathway by regulating metabolic and transcriptomic profiles and thus contribute to the regulation of colonic inflammation.

### 2.6. Regulates Colon Motility

Colon motility is a term to describe the peristaltic motion of the colon during the transportation of stool from small intestine to the rectum. Impaired colon motility is a frequently observed condition in patients with IBD [65] and may be an important factor that affects the development of IBD [66], possibly by regulating neuroplasticity in both active and quiescent IBD [67]. Using DSS-induced colitis model, Watanabe et al. [68] found that DSS treatment could induce neuroanatomical changes and damages to cholinergic neurons, which are closely related to impairment of colon motility. Thus, regulating colon motility may be a potential approach for treating IBD. Some studies have reported that SCFAs may have a role in modulating colon motility [69,70], although the effects are controversial. A good example for this line of evidence was provided by the study of Soret et al. [69], using ex vivo experiment the researchers investigated the effect of butyrate on the enteric nervous system (ENS) and colonic motility and found that butyrate could increase cholinergic-mediated colonic circular muscle contractile response. Regarding the mechanisms, they showed that butyrate could enhance the ratio of choline acetyltransferase (ChAT) but not neuronal nitric oxide synthase (nNOS)-immunoreactive myenteric neurons in an monocarboxylate transporter 2 (MCT2) dependent manner. While they also revealed that acetate and propionate did not have this effect. In comparison to this study, Cherbut et al. [70] found that intracolonic infusion of propionate or butyrate could significantly reduce colon motility and increase transit rate. About the mechanisms, the researchers revealed that local nerve fibers and polypeptide YY (PYY) were involved in the inhibitory role of the SCFAs since inhibition of intraluminal nerve activity using procaine infusion or neutralization of circulating PYY could block the effect of SCFAs on colon motility. Therefore, the exact role of SCFAs in colon motility may need to be further determined. The debatable effects of SCFAs in colon motility were also reported in studies of irritable bowel syndrome (IBS). As shown in Shaidullov et al.’s study [71], the researchers proposed that an imbalanced stimulatory and inhibitory effects of SCFAs on the regulation of colon contractility led to accelerated transit in IBS. Similar mechanisms in regulation of colonic motility by SCFAs may also apply to IBD and need to be validated in future.

## 3. Traditional Medicine and Their Derivatives Act on SCFAs to Alleviate Related Colitis through a Variety of Mechanisms

As mentioned above, recent studies in the literature have described that some traditional decoctions, formulations, and natural compounds can regulate the generation of SCFAs to alleviate animal models of colitis. In these studies, multiple experimental animals were treated with chemicals, such as DSS, which causes epithelial damage and subsequently allows the entry of intestinal microflora, thereby stimulating immune cells in the lamina propria to induce intestinal inflammation. The manifestations of the models to some extent mimic symptoms of IBD patients and are widely used to simulate the pathophysiological process of IBD and study the pathogenesis and molecular mechanisms of this disease. This section will discuss the effects, animal models, and related mechanisms of multiple traditional medicines, especially their roles in generating SCFAs. The detailed information is also summarized in Table 1.

### 3.1. The Major Decoctions/Compounds That Trigger the Production of SCFAs in Treating UC-Associated Colitis

#### 3.1.1. Coptis Chinensis Polysaccharides (CCP) and Berberine (BBR)

CCP and BBR both have a role in alleviating intestinal inflammatory conditions. Wang et al. [72] studied the combined effect and the mechanisms of CCP and BBR on colitis. Using DSS-induced colitis in mice, the researchers showed that the administration of CCP and BBR had an impact on suppressing inflammatory conditions in the colon, possibly by regulating the production of SCFAs and related gut bacteria, which promoted the expression of tight junction proteins by epithelial cells and activated IL-22 producing cells via modulating AhR signaling pathway. The altered SCFAs included acetate, propionate, butyrate, isobutyrate, valerate, and isovalerate. It should be noted that BBR alone could also have an anti-colitis effect as evidenced by Sun et al.’s study [73]. In comparison to the effect of combined administration, BBR alone also increased the levels of the SCFAs, but the types of the SCFAs regulated by BBR alone were different from those affected by the CCP/BBR combination. BBR alone could only up-regulate the levels of acetic acid, butanoic acid, and pentanoic acid. Moreover, CCP/BBR could considerably increase the abundance of SCFA-producing bacteria, which was found to be drastically reduced in IBD patients, including Akkermansia, Bacteroides, and Faecalibaculum. Correlation analysis revealed that Bacteroidetes and Akkermansia had strong positive correlations with the contents of SCFAs. It should be noted that Akkermansia is an intestinal mucin-degrading bacterium and can produce two kinds of SCFAs (acetate and propionate). The cross-feeding of Akkermansia and butyrate-producing bacteria could promote the levels of SCFAs. All these results suggested that promoting the abundance of SCFA-producing bacteria, thereby increasing the contents of SCFAs in the body and activating the AhR/IL-22 pathway, might be the potential synergistic mechanism of CCP and BBR [72].

#### 3.1.2. Gegen Qinlian Decoction (GQD)

An example of herbal decoctions that regulate the levels of SCFAs is Gegen Qinlian Decoction (GQD), a Chinese formula widely applied in treating inflammatory conditions of the intestine such as diarrhea. Using a diarrhea model of piglets, Liu et al. [74] reported that GQD could ameliorate diarrhea symptoms by up-regulating the abundance of SCFA-producing microorganisms such as Akkermansia and Bacteroides. These changes thus induced an increase in the three major types of fecal SCFAs, including acetic acid, propionic acid, and butyric acid, which attenuated colonic inflammatory responses by inhibiting the NF-κB pathway and HDAC. The increased commensal bacteria can also regulate goblet cell differentiation to finetune mucus production, which promotes the integrity of the intestinal mucosal barrier [75]. Of note, the role of GQD in inducing SCFAs was also observed in the treatment of type 2 diabetes, it could increase the abundance of probiotics and SCFA-producing bacteria such as Bifidobacteria [76]. Wang et al. [77] found that GQD could suppress UC by modulating the ferroptosis pathway in mice. Under this circumstance, GQD-induced production of SCFAs in the intestine might play an important regulatory role in the suppression of UC-associated conditions. Meanwhile, collecting the above information, it is reasonable to assume that the production of SCFAs may act as upstream regulators of ferroptosis. However, some recent studies in cancers revealed that some salts of SCFAs such as sodium butyrate could induce ferroptosis of various cancer cells [78,79]. In addition, SCFAs generated in response to GQD were associated with changes in oxidative stress. A piece of evidence proved that oral administration of GQD reduced oxidative stress in the colon of UC model mice, as demonstrated by the decrease of myeloperoxidase (MPO) activity and malondialdehyde (MDA) level and the increase of glutathione content [80]. Thus, besides the various beneficial effects of SCFAs on colonic inflammation, the potential effects of SCFAs on ferroptosis in colitis need to be determined in future studies.

#### 3.1.3. Baicalin

Baicalin is a flavone glycoside extracted from many herbal plants and has been shown to have inhibitory effects on UC. A study by Zhu et al. [10] investigated the effect of baicalin on the structure and composition of microbiota and the levels of SCFAs in the gut using a rat model of colitis induced by trinitrobenzene sulphonic acid (TNBS). The researchers showed that baicalin could ameliorate symptoms in the UC model via various mechanisms. The compound repressed TNBS-induced ROS and MDA, meanwhile enhanced the levels of GSH and SOD in the colon. It also suppressed the Th17/Treg ratio and up-regulated the production of mucins and tight junction proteins such as ZO-1 and Occludin. Moreover, baicalin treatment improved gut dysbiosis by changing the ratio of Firmicutes and Bacteroidetes. More importantly, the authors revealed that baicalin could up-regulate the abundance of butyrate-producing bacterial species such as *Butyricimonas* spp. and *Roseburia* spp. This finding was consistent with the increased level of butyrate in the feces. The changes in butyrate might be an upstream regulator of the intestinal changes since this SCFA has multiple regulatory roles in the restoration of gut inflammation, including its regulation of the protective proteins such as tight junction proteins and mucins, the differentiation of Th17/Treg, and pro-inflammatory cytokines.

#### 3.1.4. Qingchang Huashi Formula (QHF)

QHF is a formula widely used in treating UC-associated symptoms in traditional Chinese medicine. Hu et al. [81] studied the mechanisms of QHF in treating UC and found that QHF could inhibit DSS-induced colitis in mice. Constituents of QHF could maintain gut homeostasis by regulating gut microbiota and their metabolites and regulating the proliferation of crypt stem cells and the functions of goblet cells in the intestine. In this study, the researchers found that DSS treatment could down-regulate the levels of SCFAs, including butyrate acid, isobutyric acid, and valeric acid. In comparison, the administration of QHF significantly reversed these changes. It is worth noting that QHF treatment also significantly reduced the expression of NLRP3, IL-1β, and IL-18 on both mRNA and protein levels. Although the authors of this paper did not directly examine if the reduced NLRP3 expression was associated with the increase of SCFAs in the colon, many previous studies had shown that various SCFAs such as butyrate could suppress the activation of the NLRP3 inflammasome [82]. Thus, targeting the NLRP3 inflammasome via up-regulating the SCFAs is at least one of the mechanisms employed by QHF to exert its anti-colitis effects.

#### 3.1.5. Pulsatilla Decoction (PD)

PD is a formula widely used in treating UC. Niu et al. [83] studied the associated mechanisms of PD in treating this disease using a DSS-induced colitis model in mice and found that PD administration could induce a significant decline in the symptoms of the mic triggered by DSS. In further experiments, the researchers showed that PD could enhance the abundance of Bacteroidetes and meanwhile down-regulate the enrichment of Firmicutes and Proteobacteria. At the same time, PD treatment significantly up-regulated the levels of total fecal SCFAs, including acetate and propionate. The changes in SCFAs induced by PD were consistent with the levels of pro-inflammatory cytokines in the colon, including IL-1β, TNF-α, and IL-17. In comparison, the level of the anti-inflammatory cytokine, IL-10, was enhanced by PD administration. Similar to many other therapeutic drugs, PD also improved the production of protective proteins in the gut such as Occludin, ZO-1, Claudins, and GPR43. These observations suggest that the anti-UC effect of PD might be related to its effect on promoting SCFA production. Regarding the intracellular pathways, although the Niu et al. study did not examine the related pathways activated by PD, the suppressive effect of PD on various pro-inflammatory cytokines might indicate its potential effect on the NF-κB pathway. Moreover, a study by Wang et al. [84] disclosed that PD might also regulate the PI3K-AKT-mTORC1 signaling pathway in alleviating colitis. In addition, Li et al. [85] observed that PD could regulate the GPR43-NLRP3 pathway in the treatment of UC. Thus, multiple pathways contributed to the suppressive role of PD in colitis inhibition.

#### 3.1.6. Astragalus Membranaceus and Codonopsis Pilosula (PAC)

Tang et al. [86] studied the effects of the polysaccharides from PAC in a mouse model of colitis and showed that PAC could ameliorate symptoms of colitis and alleviate colonic injury induced by DSS. Their further studies revealed that the changes induced by PAC were achieved by activating AhR and promoting the production of SCFAs, including isovaleric acid and butyrate. However, the intracellular signaling pathways triggered by the condition of PAC treatment were not studied. Although the role of butyrate in activating various signaling pathways has been described, the combination of two types of SCFAs may trigger different signaling pathways. Thus, PAC, especially isovaleric acid and butyrate, induced intracellular signaling pathways need to be determined in the future.

#### 3.1.7. Hericium Erinaceus Mycelium (HEM)

It is interesting to note that a high concentration of acetic acid could induce colitis in rats. Using this model, Shao et al. [87] studied the potential effect of EP-1, the polysaccharide purified from HEM, on colitis and found that the rats’ exposure to EP-1 manifested reduced colonic inflammation. The researcher’s subsequent studies revealed that EP-1 treatment could change the structure of the gut microbiota and enhance the levels of SCFAs. In addition, EP-1 significantly suppressed the levels of GPR41 and GPR43, two important receptors of SCFAs, including acetic acid. Therefore, the inhibitory role of EP-1 on GPR41 and GPR43 might block the acetic acid-induced signaling pathway and thus ameliorate the colonic inflammation. Considering this possibility, EP-1-induced SCFAs might not achieve their suppressive effects on colitis via GPR41 and GPR43 since these receptors could be hampered by EP-1. Additionally, EP-1 can increase SOD activity, reduce ROS content and oxidative damage in vitro and in vivo [87], which also serve as regulator of colonic inflammation. Another study by Ren et al. [88] also evaluated the role of polysaccharides of HEM in DSS-induced colitis in mice. They found that the polysaccharide could suppress clinical manifestations of colitis by down-regulating the markers of oxidative stress such as nitric oxide (NO) and the pro-inflammatory cytokines, including IL-1β, TNF-α, and IL-6. The study also revealed that NF-κB, MAPK, and AKT signaling pathways were involved in the inhibitory effect of HEM polysaccharide on colitis. It is not sure if the two polysaccharide components of HEM in the above two studies are the same, we could speculate that EP-1-induced SCFAs might also contribute to the inhibition of NF-κB/MAPK/AKT pathways observed in Ren et al.’ study.

#### 3.1.8. Herba Origani Extract Pulvis (HOEP)

Yu et al. [89] studied the effect of HOEP on DSS-induced colitis in mice and showed that HOEP could reduce DSS-induced inflammatory response in the colon. For the mechanisms, the authors showed that HOEP had a role in regulating colonic dysbiosis and enhancing the enrichment of Bacteroidota, which produces various SCFAs such as acetate and propionate. To date, the signaling pathways triggered by HOEP have not been determined. Studies have demonstrated that HOEP can significantly reduce the levels of IL-1β and TNF-α in a dose-dependent manner, possibly by enhancing the production of SCFAs in the colon [89].

#### 3.1.9. Paeonol

Zheng et al. [90] studied the effect of Paeonol on a colitis model induced by DSS and showed that Paeonol could ameliorate UC-associated colonic symptoms. To investigate the related mechanisms, the researchers examined the effect of Paeonol on gut microbiota and intestinal metabolites. They found that Paeonol could significantly enhance the abundance of gut microbiota and alter multiple metabolites, including SCFAs.

#### 3.1.10. Huangqin Decoction (HQD)

Li et al. [91] studied the effect of Huangqin decoction HQD on DSS-induced colitis in mice and found that HQD administration could induce a decline in body weight loss, disease activity index (DAI) induced by DSS and improve the intestinal barrier function. The authors also disclosed that HQD could target the Ras-PI3K-Akt-HIF-1α and NF-κB pathways to achieve its anti-inflammatory effects. Another related study provided evidence that the signaling pathway might be inhibited by HQD-mediated production of SCFAs. In this study, Zhu et al. [92] found that HQD could enhance the abundance of Clostridium and trigger the production of butyric acid. Using in vitro studies, the researchers found that sodium butyrate (NaB) could enhance cellular apoptosis and reduce the PI3K-AKT pathway.

#### 3.1.11. Sishen Wan (SSW)

Using the DNBS-induced colitis model, Wang et al. [93] evaluated the role of SSW in treating colonic inflammation and found that SSW could attenuate DNBS-induced pathological changes in the colon by reducing pro-inflammatory cytokines and promoting anti-inflammatory cytokines. Meanwhile, SSW could enhance the levels of PPARγ and TGF-β1 and decrease the level of STAT3. Moreover, SSW could alter the ratio of Th17/Treg in the colon. These changes might be associated with the alterations in gut microbiota and their metabolites since SSW administration induced a significant enrichment of Firmicutes and a decline of Bacteroidota, which was consistent with the increased butyric acid in the feces.

#### 3.1.12. Composite Sophora Colon-Soluble Capsule (CSCC)

Chen et al. [94] found that the CSCC could attenuate inflammation of DSS-induced colitis in mice. In further studies, the researcher showed that CSCC could trigger the production of probiotics such as the Bacteroidales S24-7 genus. This result was accompanied by an increased level of butyric acid in the colon. Moreover, the administration of CSCC triggered a decrease in Lti ILC3s and an increase in NCR+ ILC3s. The change in ILC3s might lead to the production of intestinal IL-22, a critical cytokine for gut epithelial barrier function. All these findings might suggest a therapeutic potential of CSCC for UC.

#### 3.1.13. Acorn-Fed Ham

Fernández et al. [95] studied the impact of a traditional cured meat product, acorn-fed ham on UC using a DSS-induced colitis model in mice. The researchers found that in comparison to conventional vegetable rat feed, feeding with acorn-fed ham could change the composition of gut microbiota, specifically, the bacterial genera with anti-inflammatory properties showed significant enrichment and thus induced an overall reduction of parameters associated with colitis such as the disease activity, epithelial damage and the infiltration of inflammatory cells. Regarding the mechanisms, the authors showed that the acorn-fed ham up-regulated antioxidants such as oleic acid in plasma and increased short-chain fatty acids such as isobutyric, isovaleric, and valeric acids in cecum. Thus, acorn-fed ham might affect colitis by regulating the production of oleic acid and various SCFAs in the colon.

#### 3.1.14. Fermented Astragalus (FA)

Fermentation is a biotechnological method widely used in processing traditional Chinese medicine and increasing its functions. A study by Li et al. [96] evaluated the effect of FA on treating UC using a mouse colitis model induced by DSS. They identified the metabolites of FA after processing of Lactobacillus plantarum and found that FA pre-treatment could alleviate DSS-induced pathological changes, including histological lesion, colon length, DAI score, intestinal barrier damage, the levels of MPO, and IgE. Other changes induced by FA included a decrease in pro-inflammatory cytokines and an increase in anti-inflammatory cytokines. Meanwhile, FA triggered an alteration of gut microbiota and up-regulated the abundance of Akkermansia and Alistipes. The changes in gut microflora were positively correlated with the levels of SCFAs in the colon, including acetic acid, propionic acid, butyric acid, isobutyric acid, valeric acid, and isovaleric acid, which play an essential role in protecting the epithelial barrier function as evidenced by the increased levels of tight junction proteins such as ZO-1, occludin, and mucus-producing gene MUC2. Of note, the unfermented Astragalus did not have the anti-colitis effect, further highlighting the efficacy of fermentation.

#### 3.1.15. Indigo Naturalis

Indigo naturalis is a traditional medicine widely used in treating UC. Sun et al. [97] investigated the mechanisms of Indigo naturalis in treating UC using a rat model induced by DSS. They showed that the protective effect of Indigo naturalis was associated with its role in regulating gut microbiota since depletion of microbiota using antibiotics blocked the inhibitory role on colitis. More importantly, the administration of indigo naturalis could enhance fecal butyrate levels. This change correlated with the up-regulated enrichment of Ruminococcus_1 and Butyricicoccus. Further tests disclosed that indigo naturalis could improve the expression of receptors of SCFAs such as GPR41 and GPR43.

#### 3.1.16. Schisandra Chinensis Polysaccharide (SCP)

Su et al. [98] studied the role of Schisandra chinensis Polysaccharide (SCP) on colitis using a DSS-induced colitis model in mice. They showed that SCP significantly improved the damaged epithelium triggered by DSS. The role of SCP might be associated with its impact on gut microbiota and fecal SCFAs since SCP enhanced levels of butyric acid, isobutyric acid, and valeric acid, which also showed a correlation with the enrichment of particular species of gut microbiota.

#### 3.1.17. Pulsatilla Chinensis Saponins (PCS)

PCS is a bioactive ingredient of Pulsatilla decoction, a traditional medicine used in treating UC. A study by Li et al. [85] showed that PCS exerted its anti-colitis role by regulating SCFAs in colon tissues. Meanwhile, it also increased the level of SCFA receptor GPR43. These changes mediated by PCS might be responsible for the subsequent inhibition of the NLRP3 inflammasome and related pro-inflammatory cytokines including IL-1β.

#### 3.1.18. Galangin

Galangin is a bioactive component of Alpinia officinarum (galangal), an herbal plant rich in some specific bacterial groups that can promote SCFA production. Galangal treatment can reverse the DSS-induced reduction of SCFAs by increasing acetate and butyrate levels, possibly associated with the SCFA-producing bacteria it contains such as Butyricobacter, which might play a role in remodeling the gut microbiome [99]. To further study if the role of galangal was mediated by its major bioactive compound, galanin, Xuan et al. [99] employed a DSS-induced colitis model and evaluated the role of galangin in colonic inflammation. The researchers found that treatment with galangin reduced colonic damages triggered by DSS by suppressing colonic pro-inflammatory mediators, including IL-1β, TNF-α, IL-6, and MPO. Meanwhile, galangin could enhance autophagy and enrich and diversity of the gut microbiota. These changes were accompanied by up-regulation of SCFAs in the colon, which was associated with enrichment of *Lactobacillus* spp. and *Butyricimonas* spp. Thus, this study provided evidence that the anti-colitis effect of galangal was mediated by galangin since they induced similar beneficial alterations in animals with colitis and the study further highlighted the role of SCFAs in ameliorating colonic inflammation.

#### 3.1.19. Pinocembrin (PIN)

The PIN is a bioactive flavonoid with a potent effect on maintaining gastrointestinal homeostasis. To study the related mechanisms, Hu et al. [100] studied its impact on DSS-induced colitis in rats. DSS-treated rats showed remarkable histological damages, while these changes were significantly reduced by PIN treatment, which also had a suppressive role in the production of pro-inflammatory cytokines and improved levels of tight junction proteins. It should be noted that PIN could also restore the DSS-induced loss of SCFAs, including acetate and butyrate, and the levels were negatively correlated with disease activity, indicating an increase of SCFA-producing microbes in the gut.

In general, the chemical composition and specific role of some traditional drugs or natural compounds have not yet been fully determined. The detailed list of SCFAs induced by these traditional medicines and the molecular mechanisms and signaling pathways triggered by the SCFAs still need to be identified. Further research to solve these problems may help the development of new drugs for the treatment of IBD by regulating SCFAs.

**Table 1:** Traditional medicine-derived decoctions and compounds alleviate colitis by modulating SCFAs.

No.	Decoction	Constituent	Mechanism	Regulated SCFAs	Pathway	Dose/Concentration	Disease Model	Reference
(1)	Baicalein (Baicalin)	5,6,7-trihydroxyflavone-7-b-d-glucuronate	Promote the level of butyrate in SCFAs, regulate protective proteins such as tight junction protein and mucin, balance the ratio of Th17/Treg, and inhibit pro-inflammatory cytokines	Butyric acid	NF-κB	25.0, 50.0, and 100 mg/kg by gavage every 2 days for 14 days	TNBS-induced colitis	[10]
(2)	Coptis chinensis polysaccharides and berberine (CCP/BBR)	Polysaccharides (CCP) and berberine (BBR)	Increases the abundance of SCFA-producing bacteria and increases the level of SCFAs	Acetic acid, propionic acid, butyric acid, isobutyric acid, valeric acid, and isovaleric acid	AhR/IL-22	15 mg/kg (CCP), 50 mg/kg (BBR), once a day for 10 days	1.5% DSS-induced colitis in mice	[72]
(3)	Berberine	Isoquinoline alkaloids	Increase the level of SCFAs	Acetic acid, butyric acid, and valeric acid	STAT3/NF-κB	25 mg/kg BBR in 2% γ-cyclamycin solution once a day for one week	3% DSS-induced colitis in mice	[73]
(4)	Gegen Qinlian Decoction (GQD)	Flavonoids *C*-glycosides, flavonoids *O*-glucosides, phenylisoquinoline alkaloids, free flavonoids, flavonoids *O*-glycosides, coumarins, triterpenoid saponins, etc.	Increases the abundance of SCFA-producing bacteria such as *bifidobacterium*, reduces colon inflammatory responses, and promotes the integrity of the intestinal barrier	Acetic acid, propionic acid, and butyric acid	TLR/NF-κB/HDAC	GQD tablet extract (oral, 49 g/piglet, once a day for one week)	Oral *E. coli* induced diarrhea in piglets	[74]
(5)	Qingchang Huashi Formula (QHF)	Astragalus, Paeoniae paeoniae (Peony), Pulsatilla, Angelica dahurica and dried sausage, Scutellaria baicalensis, jade and so on	Upregulation of SCFAs content inhibits the activation of NLRP3 inflammasome, reduces the production of proinflammatory cytokines in colon, and maintains a stable intestinal environment	Butyric acid, isobutyric acid, and valeric acid	NLRP3/IL-1β	18 g/kg, 3 days before DSS treatment until the end of the experiment	2.5% DSS-induced acute colitis in mice/ 2% DSS-induced chronic colitis in mice	[81]
(6)	Pulsatilla decoction (PD)	Aesculin, aesculin, baicalin hydrochloride, palmatine chloride, berberine, Pulsatilla pulsatilla saponin B4	Induces levels of SCFAs, and improves the production of protective proteins in the intestine, such as Occludin, ZO-1, Claudins, and GPR43	Acetate and propionate	NF-κB; PI3K-AKT-mTORC1, NLRP3	4.2, 7.5, and 8.1 g/kg by gavage once daily for 7 days	3% DSS-induced colitis in mice	[83]
(7)	Pulsatilla chinensis saponin (PRS)	Pulsatilla triterpenoid saponins	Increases the levels of SCFA receptor GPR43 and inhibits the NLRP3 inflammasome and related pro-inflammatory cytokines	Unidentified	NLRP3	300 mg/kg, orally, on the 10th day	DSS-colitis in rats (4 g/kg/d) for 10 days	[85]
(8)	Polysaccharides from Astragalus membranaceus and Codonopsis pilosula (PAC)	Mannose, ribose, rhamnose, glucuronic acid, glucose, xylose, arabinose, etc	Activate the aromatic hydrocarbon receptor (AhR) and promote SCFAs production	Isovaleric acid and butyric acid	Unidentified	300, 600 mg/kg/day	2.5% DSS-induced colitis in mice	[86]
(9)	Polysaccharide of Hericium erinaceus mycelium (HEM)	The polysaccharide component EP-1 is composed of glucose, mannose and galactose	Alter the structure of the gut microbiota, increases the level of SCFAs, inhibits the levels of GPR41 and GPR43	acetic acid	NF-κB /MARK /AKT	0.6, 1.2 g/kg, once daily for 10 days	4% acetic acid-induced colitis in mice	[87,88]
(10)	Herba Origani Extract Pulvis (HOEP)	Rosmarinic acid, thymol, and carvacrol	Increase the production of SCFAs, reduce the production of pro-inflammatory cytokines, and maintain a stable intestinal environment	Acetic acid and propionic acid	Unidentified	0.125, 0.25, 0.5, 1 g/kg of HOEP, orally, once a day for 10 days	3% DSS-induced colitis in mice	[89]
(11)	Paeonol (Pae)	2′-hydroxy-4′-methoxyacetophenone; C_9_H_10_O_3_	Increased gut microbiota abundance with SCFAs content, altered various metabolites	Unidentified	Unidentified	50, 100, and 500 mg/kg of Pae twice daily for 7 days	3% DSS-induced colitis in mice	[90]
(12)	Huangqin Decoction (HQD)	117 active compounds	Increase the level of SCFAs	Increases the abundance of *clostridium*, butyric acid	PI3K/Akt	2.275, 4.55, and 9.1 g/kg once daily via gavage for 7 days	3% DSS-induced colitis in mice	[91,92]
(13)	Sishen Wan (SSW)	Psorus, Evodia, nutmeg, schisandra, jujube and so on	Changes in the Th17/Treg ratio in the colon increase the abundance of SCFAs producing bacteria	Butyric acid	Th17/Treg balance	6 g, 12 g/kg in distilled water (10 mL/kg) by gavage once a day for 21 days	DNBS-induced colitis in rats	[93]
(14)	Composite Sophora colon-soluble Capsule (CSCC)	Angelica powder, green Dai, matrine, ulmus and licorice, etc	Increase the level of SCFAs	Butyric acid	AKT-STAT3	0.5, 3.84 g/kg/d, until day 14	3% DSS-induced colitis in mice	[94]
(15)	Acorn-fed ham	Unidentified	Up-regulates the plasma antioxidants such as oleic acid and Increase the level of SCFAs	Isobutyric acid, isovaleric acid, and valeric acid	Unidentified	25 g ham to each cage daily, for seven days	3% DSS-induced colitis in mice	[95]
(16)	Fermented astragalus (FA)	11 different metabolites such as raffinose, progesterone and uridine	Changes the composition of gut microbiota and Increase the level of SCFAs	Acetic acid, propionic acid, butyric acid, isobutyric acid, valeric acid, and isovaleric acid	Unidentified	5 g/kg/day, FA, once a day for 7 days	3.5% DSS-induced colitis in mice	[96]
(17)	Indigo naturalis	Unidentified	Increasing SCFAs in faeces increases the abundance of SCFAs producing bacteria and increases the expression of SCFAs receptors such as GPR41 and GPR43	Butyric acid	AhR/IL-22 and NLRP3	600 mg/kg once a day for 7 days	4.5% DSS-induced colitis in mice	[97]
(18)	Schisandra chinensis polysaccharide (SCP)	Schisandra crude polysaccharide	Increase the level of SCFAs	Butyric acid, isobutyric acid, and valeric acid	NF-κB	8.0 g/kg, SCP, daily for 10 days	3% DSS-induced colitis in mice	[98]
(19)	Galangin	3,5,7-trihydroxyflavones	Increases SCFA-producing bacteria such as *Bacillus butyricum*, enhances the autophagy, enrichment, and diversity of the gut microbiota, inhibits colonic pro-inflammatory mediators, colonic inflammation	Acetic acid and butyric acid	Unidentified	15 mg/kg, orally once daily for 7 days	3% DSS-induced colitis in mice	[99]
(20)	Pinocembrin (PIN)	Pinot umbrella protein	Increases the levels of SCFAs and SCFA-producing microbes	Acetic and butyric acid	NF-κB	5 and 10 mg/kg, orally, once a day for 7 days	3.5% DSS-induced colitis in mice	[100]

### 3.2. The Common Mechanisms of SCFAs Derived from Traditional Medicine in Alleviating Colitis

**Figure 2:**
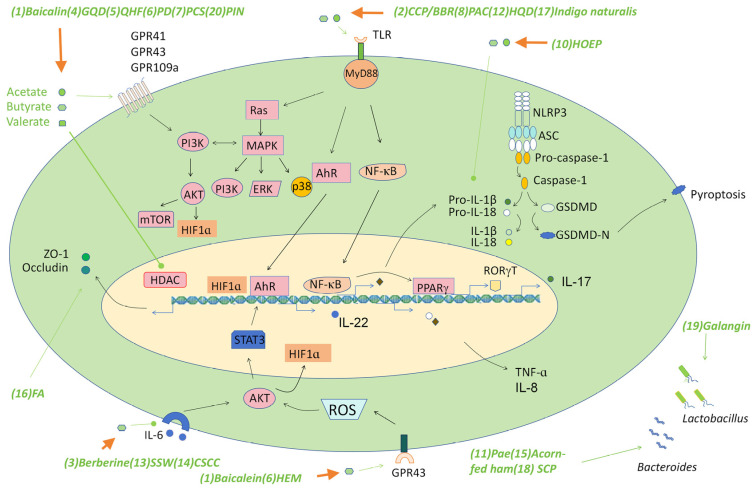
The signaling pathways triggered by traditional medicine-derived SCFAs. Some decoctions/compounds in traditional Chinese medicines alleviate colitis by inducing the production of various SCFAs such as acetate, butyrate, and valerate, which target several signaling pathways such as GPRs to trigger the production of various barrier-supporting proteins such as the tight junction proteins, mucins, and immunomodulatory cytokines such as IL-22, which play a critical role in maintaining gut homeostasis. SCFAs also suppress pro-inflammatory pathways such as the TLR-NF-κB pathway, the NLRP3 inflammasome, and IL-6 mediated AKT pathway to down-regulate the production of pro-inflammatory cytokines such as TNF-α, IL-1β, and IL-8, which may influence IBD by regulating the recruitment of multiple immune cells to the site of inflammation. SCFAs may promote PPARγ production via the NF-κB pathway, then suppress the level of RORγT to control Th17 cell differentiation and interfere with inflammation by regulating the balance of Th17/Treg cells. Regulation of the AKT/STAT3 signaling pathway through SCFA production is an important mechanism in the treatment of colon inflammation by some traditional drugs. Abbreviations: (1) Baicalein; (2) Polysaccharides (CCP) and berberine (BBR); (3) Berberine; (4) Gegen Qinlian Decoction (GQD); (5) Qingchang Huashi Formula (QHF); (6) Pulsatilla decoction (PD); (7) Pulsatilla chinensis saponin (PCS); (8) Polysaccharides from Astragalus membranaceus and Codonopsis pilosula (PAC); (9) Polysaccharide of Hericium erinaceus mycelium (HEM); (10) Herba Origani Extract Pulvis (HOEP); (11) Paeonol (Pae); (12) Huangqin Decoction (HQD); (13) Sishen Wan (SSW); (14) Composite Sophora colon-soluble Capsule (CSCC); (15) Acorn-fed ham; (16) Fermented astragalus (FA); (17) Indigo naturalis; (18) Schisandra chinensis polysaccharide (SCP); (19) Galangin; (20) Pinocembrin (PIN).

As described above, many traditional medicine can ameliorate symptoms of colitis in several animal models by regulating the production of SCFAs. The effects of the SCFAs are achieved through several mechanisms, mainly including regulating Th17/Treg cell balance or suppressing a number of signaling pathways such as the NF-κB, the RORγT, the AKT-STAT3, NLRP3 inflammasome, and the AhR signaling pathways. Here, we discuss these new findings and the associated mechanisms triggered by various SCFAs (Figure 2 and Table 1). On this basis, we provide our understandings of future studies that may lead to development of SCFA-based anti-IBD drugs.

#### 3.2.1. NF-κB

NF-κB is a key transcription factor involved in many biological processes and its excessive activation may affect the development of IBD through various mechanisms. NF-κB signaling pathway is the most common target of many herbal decoctions or natural compounds. As mentioned above, the effect of GQD on inhibiting the expression of inflammatory cytokines was mediated by suppressing the TLR4-mediated NF-κB signaling pathway [80]. The effect of GQD was associated with the inhibition of HDAC by SCFAs [48,101]. GQD-derived SCFAs could also stabilize the expression of IκBα and attenuate the level of NF-κB-P65 [102,103,104]. The second example for targeting NF-κB pathway was provided by the study of baicalin, which alleviates DSS-induced chronic UC, possibly by SCFA-mediated inhibition of NF-κB activation and subsequent expression of IL-33 [105]. Moreover, some polysaccharides could suppress the NF-κB pathway to exert their anti-colitis effect. A good example of this line of evidence is provided by SCFAs derived from Astragalus polysaccharides, which attenuate colonic inflammation by suppressing NF-κB activation and release of related pro-inflammatory cytokines [106,107]. Furthermore, NF-κB is also the target of SCFAs generated during treatment of SCP or BBR in inhibiting colonic inflammation [73,98]. Thus, the NF-κB signaling pathway is a common target of SCFAs derived from many decoctions and compounds in traditional medicine.

#### 3.2.2. Th17/Treg Balance

T helper 17 (T17)/Regulatory T (Treg) imbalance is a common inflammation-related mechanism. More and more evidence shows that the destruction of Th17/Treg balance is considered to be an important mediator of tissue damage in UC colonic mucosal ulcer [93], and also an important factor in the pathogenesis of IBD [10]. IL-6-induced signal transducer and activator of transcription 3 (STAT3) signaling plays a key role in the differentiation of CD4+ T cells to Tregs or Th17 cells, which determines the quiescence or the development of colonic inflammation [108]. SCFAs can regulate the homeostasis of colonic Treg cells and activate the intestinal mucosal immune system [109]. Studies have found that the traditional drug SSW increases the concentration of butyric acid in the intestine by increasing the expression of PPAR-γ and correcting the imbalance of the Treg/T17 immune axis [93]. Furthermore, SCFA, especially butyrate, was found in Astragalus and Codonopsis polysaccharide (AERP CERP), which can regulate the differentiation of T cells and the secretion of inflammatory factors, indicating that they may have potential value in the recovery of UC [110]. Moreover, Baicalin reduced the ratio of Th17/Treg in TNBS-induced UC rats [111]. An increase in the ratio of Th17/Treg cells was observed in the UC group, and baicalin treatment inhibited this induction, indicating that baicalin attenuated inflammation. In addition, FA supplementation can also interfere with the inflammatory state by regulating the balance of Th17/Treg-related cytokines. It changed the structure of the intestinal microflora and enriched the abundance of Akkermansia and Alistipes, which were positively correlated with the production of SCFA [96]. Thus, regulating the balance of Th17/Treg is an important mechanism of traditional medicine-derived SCFAs in treating UC-associated symptoms.

#### 3.2.3. RORγT

RORγT is a transcription factor that controls the differentiation of Th17 cells and is essential for the secretion of Th17 effector cytokines [93]. Previous studies have shown that Th17 cells may infiltrate the inflammatory bowel of UC patients and produce effector cytokines to regulate colonic inflammation. It has been shown that the intestinal microflora of IBD patients can affect Th17 cell differentiation in the intestine of mice [112]. The intestinal microbiota can activate the expression of PPAR-γ in epithelial cells of the intestine and maintain intestinal mucosal homeostasis [113]. This effect may be mediated by microbiota-derived SCFAs, which regulate the differentiation direction of CD4+ T cells, restore the balance of the immune axis, and inhibit the inflammatory response [93]. Some recent studies have also shown that traditional medicine decoction regulates RORγT activity [114]. As described above, administration of SSW enhances the production of PPAR-γ in the colon. The activation of PPAR-γ has a protective effect on colitis, and its expression is reduced in UC patients [113]. Activation of PPAR-γ reduces the production of local intestinal IL-6 and inhibits the elimination of the co-repressor SMRT from the Rorc promoter, thereby inhibiting RORγT production and subsequent Th17 cell differentiation [115]. According to previous studies, the expression of TGF-β1 and PPAR-γ in colon tissue treated with SSW was significantly increased, and the expression of STAT3 and p-STAT3 was significantly decreased. SSW can increase the concentration of butyrate in the intestinal microflora, which can reduce intestinal permeability, enhance the intestinal mucosal barrier, down-regulate the expression of pro-inflammatory cytokine IL-6 [8], increase the expression of PPAR-γ, inhibit the production of inflammatory cytokines, improve the damaged intestinal barrier function under inflammatory conditions, thereby suppressing the disrupted Th17 response and reducing the severity of UC [103,116]. Therefore, regulating the expressions of PPAR-γ and RORγT is an essential approach of SSW in treating UC by enhancing the production of SCFAs.

#### 3.2.4. AKT-STAT3

The AKT-STAT3 pathway is an intracellular signaling pathway that has a significant impact on many biological processes, such as cell survival, proliferation, and angiogenesis. Activation of this pathway has a significant impact on UC [94]. In traditional medicine, some drugs can regulate the intestinal barrier to alleviate UC-related symptoms by regulating the AKT-STAT3 pathway [117]. CSCC provides an example of this evidence. Researchers have found that SCFAs promote NCR+ ILC3 proliferation and IL-22 production by activating the downstream AKT/STAT3 signaling pathway, leading to intestinal epithelial cell regeneration, AMP production, and intestinal epithelial cell mucus secretion [94]. Thus, regulating the AKT/STAT3 signaling pathway via generated SCFAs is an important mechanism employed by some traditional medicines in treating colonic inflammation. However, the components of the traditional drugs in promoting SCFA production need to be further determined.

#### 3.2.5. NLRP3 Inflammasome

The NLRP3 inflammasome is a protein complex that plays a critical role both in host defense against infections and in the regulation of autoinflammatory diseases. Some recent studies showed that the components of the NLRP3 inflammasome showed increased expression in IBD patients and thus play a role in regulating the development of IBD [118]. Some SCFAs enter the blood through the intestine and bind to GPR43 on the surface of immune cells to affect the activation of the NLRP3 inflammasome. With the increase of SCFA content, the natural sensor GPR43 was activated, and the protein level of GPR43 was up-regulated. This inhibits the activation of NLRP3 inflammasome and thus reduces the production of pro-inflammatory cytokines including IL-1β and IL-18 in the colon, maintains a stable intestinal environment, reduces the permeability of intestinal mucosa, and promotes the recovery of the intestinal barrier [119]. It was found that the GPR41/43 pathway is necessary to prevent indigo-induced DSS-induced colitis, and the change of indigo-induced microflora may be a key regulator for this effect by increasing the production of SCFAs, especially butyric acid [97], which suppresses the activation of GPR43-NLRP3 signaling pathway and reduces the level of pro-inflammatory cytokines, thereby improving DSS-induced colitis symptoms.

#### 3.2.6. AhR Pathway

The AhR is transcription factor located in the cytoplasm. It can be activated by various ligands derived from both exogenous and endogenous origins. After binding to the ligands, the AhR complex enters the nucleus and causes the expression of the target genes [120]. The activation of AhR can induce IL-22 production, enhance enzyme metabolism, and lead to suppression of immune response and promotion of mucosal healing. Previous studies found that the expression levels of AhR and several AhR ligands were decreased in inflamed colon tissues of IBD patients [121,122], indicating a potential role of AhR signaling pathway in the development of IBD, which might act as a treating target for IBD. Several recent studies have shown that traditional medicine derived SCFAs could improve colonic inflammation by regulating the activity of AhR. For instance, in the DSS-induced colitis model, administration of indigo had a role in preventing intestinal inflammation [123]. Using AhR deficient mice, the researchers showed that the effect of indigo was dependent on the activation of AhR, which up-regulated the levels of the anti-inflammatory cytokines, including IL-10 and IL-22. However, the exact target of the SCFAs in AhR signaling pathway has not been identified. In the same line of evidence, Wang et al. [72] showed that the level of AhR was significantly increased in colitis mice treated with BBR and CCP, which mediated the increased abundance of SCFA-producing bacteria. The later enhanced the production of SCFAs in the gut and subsequently up-regulated the production and activation of AhR and its downstream target molecule, CYP1A1. Activated AhR and CYP1A1 mediated IL-22 production to mitigate colonic inflammation. Thus, activating AhR pathway is a critical mechanism employed by SCFAs to achieve the anti-colitis activity.

## 4. Limitations and Solutions

An increasing number of studies have provided evidence that numerous decoctions or formulas in traditional medicine have promising therapeutic effects on colitis in animal models, at least in part, by regulating the production of various SCFAs. While further development and application of these medicines in clinical practice are still limited by several challenges: (1) The exact ingredients or compounds that play the role of promoting the production of SCFAs in a decoction/formula need to be identified since many unrelated ingredients may enhance the toxicity of the effective compounds. (2) The SCFAs induced by traditional medicine, or some compounds were generated by particular SCFA-producing bacteria, and thus it is important to evaluate the relative efficacy of the compounds, SCFA-producing bacteria, and the SCFAs, in in vivo studies, to determine the best approach for drug delivery. (3) Elucidating the multifaceted molecular processes behind the anti-inflammatory activity of SCFAs has been challenging because they may work with different signaling compounds. Since the role of SCFAs is sometimes combinatorial, diverse, and indirect, future research must explain its clinical treatment prospects. (4) After identification of the effective components for promoting SCFAs, their efficacy and cytotoxicity also need to be extensively evaluated in pre-clinical in vivo studies with multiple animal species. (5) The current research mainly focuses on the regulation of intestinal microbial diversity, structure, and abundance by traditional drugs, the potential mechanisms of action have not been elucidated. (6) The quality of the traditional medicines, especially some herbs, can be greatly affected by a variety of environmental factors, such as the temperature and humidity. Therefore, the examination of the chemical ingredients of the traditional medicines is necessary to guarantee the efficacy.

Given the beneficial effect of SCFAs described in numerous studies using many IBD mouse models [124], it should be noted that SCFAs may also have a pro-inflammatory effect [54]. Thus, it might be important to evaluate the status of the microenvironment before the application of SCFAs or SCFA-producing bacteria in treating UC-associated conditions. Future research must establish different standard animal models based on the relationship between the therapeutic efficacy of traditional medicine and the structure, composition, and action factors of SCFAs. In addition, it is important to further identify the composition of the decoction and its immune effect on SCFAs, which is an important issue in UC treatment research. The research work can provide a clinical basis for the applications of different traditional drugs to treat different types of UC, meanwhile largely improving the therapeutic efficacy of UC and achieving precision medicine. Additionally, integrated applications of various state-of-the-art technologies such as single-cell sequencing, multi-omics, computerized screening, microbiota interference, gene knockout techniques, and multicolor immunofluorescence in in vitro and in vivo experiments might be helpful for the identification of effective compounds in decoctions/formulas and the evaluation of the efficacy and cytotoxicity of the ingredients of traditional medicines, as well as the relative contributions of SCFAs. On this basis, a framework can be established for predicting and identifying IBD biomarkers and targets of host–microbial interactions, as well as improving the accuracy for clinical applications of the traditional medicines.

## 5. Conclusions

In this study, we summarized the major advances in the literature as to the roles of many decoctions, formulas, or compounds derived from traditional medicine and the medicines-triggered production of SCFAs in several animal models of colitis induced by various chemicals such as DSS, TNBS, DNBS, and acetic acid. In general, the traditional medicines described in this study commonly trigger the alterations of the gut microbiota of the animals, which show increased enrichment with SCFA-producing bacteria. The latter produces a variety of SCFAs in the intestine, such as acetate, propionate, butyrate, and valerate. These SCFAs in turn activate several receptors such as GPR41 and GPR43 to achieve their suppressive roles to many intracellular signaling pathways, including the NF-κB, AKT-STAT3, RORγT, NLRP3, and AhR signaling pathways. The altered activity of these signaling pathways then suppresses colon inflammation and enhances intestinal healing by regulating multiple biological processes, mainly including up-regulating the expression of barrier-supporting proteins such as the tight junction proteins, mucin, AMPs, and secretory IgAs to repair the intestinal mechanical barrier and the chemical barrier, promoting the proliferation of multiple intestinal epithelial cells, suppressing the production of ROS, inhibiting inflammatory responses in the colon, regulating colon motility, and balancing the gut microbiota and related metabolites. Overall, the SCFAs induced by various traditional medicines have provided an anti-colitis approach for the management of UC by various mechanisms.

Current studies have revealed the important role of SCFAs in body metabolism and the therapeutic potential for various related diseases. It is generally believed that SCFAs exerts beneficial effects on the host, but in certain cases, excessive SCFAs will harm the host, so the interaction between SCFAs and the host needs further research, which may promote our understanding of the specific mechanisms of action in related diseases and more effectively achieve personalized treatment. Moreover, the reduction of particular SCFA-producing bacteria in gut microbiota may be helpful to identify different subtypes of UC. The characteristic bacteria are likely to be used as diagnostic biomarkers for UC, suggesting that the usage of herbal medicine-based plant drugs may be a promising strategy for UC treatment via regulating intestinal microbiota [125]. Our study emphasizes the importance of SCFAs in maintaining intestinal health and encourages a further extensive study of traditional medicines that induce the production of SCFAs and regulate the enrichment of SCFA-producing microflora in the context of colonic inflammation, which may facilitate the development of new IBD therapies.

## Data Availability

The original contributions presented in this study are included in the article. Further inquiries can be directed to the corresponding author.

## References

[1] Guan Q. (2019). A Comprehensive Review and Update on the Pathogenesis of Inflammatory Bowel Disease. J. Immunol. Res..

[2] Stokkers P.C., Hommes D.W. (2004). New cytokine therapeutics for inflammatory bowel disease. Cytokine.

[3] Xie F., Xiong Q., Li Y., Yao C., Wu R., Wang Q., Luo L., Liu H., Feng P. (2022). Traditional Chinese Medicine Regulates Th17/Treg Balance in Treating Inflammatory Bowel Disease. Evid. Based Complement. Altern. Med..

[4] Russo E., Giudici F., Fiorindi C., Ficari F., Scaringi S., Amedei A. (2019). Immunomodulating Activity and Therapeutic Effects of Short Chain Fatty Acids and Tryptophan Post-biotics in Inflammatory Bowel Disease. Front. Immunol..

[5] Blaak E.E., Canfora E.E., Theis S., Frost G., Groen A.K., Mithieux G., Nauta A., Scott K., Stahl B., van Harsselaar J. (2020). Short chain fatty acids in human gut and metabolic health. Benef. Microbes.

[6] Vogt S.L., Peña-Díaz J., Finlay B.B. (2015). Chemical communication in the gut: Effects of microbiota-generated metabolites on gastrointestinal bacterial pathogens. Anaerobe.

[7] Louis P., Hold G.L., Flint H.J. (2014). The gut microbiota, bacterial metabolites and colorectal cancer. Nat. Rev. Microbiol..

[8] Parada Venegas D., De la Fuente M.K., Landskron G., González M.J., Quera R., Dijkstra G., Harmsen H.J.M., Faber K.N., Hermoso M.A. (2019). Short Chain Fatty Acids (SCFAs)-Mediated Gut Epithelial and Immune Regulation and Its Relevance for Inflammatory Bowel Diseases. Front. Immunol..

[9] Heimerl S., Moehle C., Zahn A., Boettcher A., Stremmel W., Langmann T., Schmitz G. (2006). Alterations in intestinal fatty acid metabolism in inflammatory bowel disease. Biochim. Biophys. Acta.

[10] Zhu L., Xu L.Z., Zhao S., Shen Z.F., Shen H., Zhan L.B. (2020). Protective effect of baicalin on the regulation of Treg/Th17 balance, gut microbiota and short-chain fatty acids in rats with ulcerative colitis. Appl. Microbiol. Biotechnol..

[11] Huda-Faujan N., Abdulamir A.S., Fatimah A.B., Anas O.M., Shuhaimi M., Yazid A.M., Loong Y.Y. (2010). The impact of the level of the intestinal short chain Fatty acids in inflammatory bowel disease patients versus healthy subjects. Open Biochem. J..

[12] Liu P., Wang Y., Yang G., Zhang Q., Meng L., Xin Y., Jiang X. (2021). The role of short-chain fatty acids in intestinal barrier function, inflammation, oxidative stress, and colonic carcinogenesis. Pharmacol. Res..

[13] Wang B., Gong Z., Zhan J., Yang L., Zhou Q., Yuan X. (2020). Xianglian Pill Suppresses Inflammation and Protects Intestinal Epithelial Barrier by Promoting Autophagy in DSS Induced Ulcerative Colitis Mice. Front. Pharmacol..

[14] Fukuda S., Toh H., Hase K., Oshima K., Nakanishi Y., Yoshimura K., Tobe T., Clarke J.M., Topping D.L., Suzuki T. (2011). Bifidobacteria can protect from enteropathogenic infection through production of acetate. Nature.

[15] Singh N., Gurav A., Sivaprakasam S., Brady E., Padia R., Shi H., Thangaraju M., Prasad P.D., Manicassamy S., Munn D.H. (2014). Activation of Gpr109a, receptor for niacin and the commensal metabolite butyrate, suppresses colonic inflammation and carcinogenesis. Immunity.

[16] Chiang H.Y., Lu H.H., Sudhakar J.N., Chen Y.W., Shih N.S., Weng Y.T., Shui J.W. (2022). IL-22 initiates an IL-18-dependent epithelial response circuit to enforce intestinal host defence. Nat. Commun..

[17] Deleu S., Arnauts K., Deprez L., Machiels K., Ferrante M., Huys G.R.B., Thevelein J.M., Raes J., Vermeire S. (2023). High Acetate Concentration Protects Intestinal Barrier and Exerts Anti-Inflammatory Effects in Organoid-Derived Epithelial Monolayer Cultures from Patients with Ulcerative Colitis. Int. J. Mol. Sci..

[18] Peng L., He Z., Chen W., Holzman I.R., Lin J. (2007). Effects of butyrate on intestinal barrier function in a Caco-2 cell monolayer model of intestinal barrier. Pediatr. Res..

[19] Capaldo C.T., Powell D.N., Kalman D. (2017). Layered defense: How mucus and tight junctions seal the intestinal barrier. J. Mol. Med..

[20] Otani T., Furuse M. (2020). Tight Junction Structure and Function Revisited. Trends Cell Biol..

[21] Tsukita S., Tanaka H., Tamura A. (2019). The Claudins: From Tight Junctions to Biological Systems. Trends Biochem. Sci..

[22] Van Itallie C.M., Anderson J.M. (2018). Phosphorylation of tight junction transmembrane proteins: Many sites, much to do. Tissue Barriers.

[23] Saleri R., Borghetti P., Ravanetti F., Cavalli V., Ferrari L., De Angelis E., Andrani M., Martelli P. (2022). Effects of different short-chain fatty acids (SCFA) on gene expression of proteins involved in barrier function in IPEC-J2. Porc. Health Manag..

[24] Miao W., Wu X., Wang K., Wang W., Wang Y., Li Z., Liu J., Li L., Peng L. (2016). Sodium Butyrate Promotes Reassembly of Tight Junctions in Caco-2 Monolayers Involving Inhibition of MLCK/MLC2 Pathway and Phosphorylation of PKCβ2. Int. J. Mol. Sci..

[25] Willemsen L.E., Koetsier M.A., van Deventer S.J., van Tol E.A. (2003). Short chain fatty acids stimulate epithelial mucin 2 expression through differential effects on prostaglandin E(1) and E(2) production by intestinal myofibroblasts. Gut.

[26] Le Poul E., Loison C., Struyf S., Springael J.Y., Lannoy V., Decobecq M.E., Brezillon S., Dupriez V., Vassart G., Van Damme J. (2003). Functional characterization of human receptors for short chain fatty acids and their role in polymorphonuclear cell activation. J. Biol. Chem..

[27] Huus K.E., Bauer K.C., Brown E.M., Bozorgmehr T., Woodward S.E., Serapio-Palacios A., Boutin R.C.T., Petersen C., Finlay B.B. (2020). Commensal Bacteria Modulate Immunoglobulin A Binding in Response to Host Nutrition. Cell Host Microbe.

[28] Wu W., Sun M., Chen F., Cao A.T., Liu H., Zhao Y., Huang X., Xiao Y., Yao S., Zhao Q. (2017). Microbiota metabolite short-chain fatty acid acetate promotes intestinal IgA response to microbiota which is mediated by GPR43. Mucosal Immunol..

[29] Brown A.J., Goldsworthy S.M., Barnes A.A., Eilert M.M., Tcheang L., Daniels D., Muir A.I., Wigglesworth M.J., Kinghorn I., Fraser N.J. (2003). The Orphan G protein-coupled receptors GPR41 and GPR43 are activated by propionate and other short chain carboxylic acids. J. Biol. Chem..

[30] Takeuchi T., Miyauchi E., Kanaya T., Kato T., Nakanishi Y., Watanabe T., Kitami T., Taida T., Sasaki T., Negishi H. (2021). Acetate differentially regulates IgA reactivity to commensal bacteria. Nature.

[31] Yamamoto Y., Morozumi T., Takahashi T., Saruta J., To M., Sakaguchi W., Shimizu T., Kubota N., Tsukinoki K. (2020). Faster Short-Chain Fatty Acid Absorption from the Cecum Following Polydextrose Ingestion Increases the Salivary Immunoglobulin A Flow Rate in Rats. Nutrients.

[32] Chai L., Luo Q., Cai K., Wang K., Xu B. (2021). Reduced fecal short-chain fatty acids levels and the relationship with gut microbiota in IgA nephropathy. BMC Nephrol..

[33] Tominaga K., Tsuchiya A., Mizusawa T., Matsumoto A., Minemura A., Oka K., Takahashi M., Yosida T., Kawata Y., Takahashi K. (2021). Evaluation of intestinal microbiota, short-chain fatty acids, and immunoglobulin a in diversion colitis. Biochem. Biophys. Rep..

[34] Milani C., Duranti S., Bottacini F., Casey E., Turroni F., Mahony J., Belzer C., Delgado Palacio S., Arboleya Montes S., Mancabelli L. (2017). The First Microbial Colonizers of the Human Gut: Composition, Activities, and Health Implications of the Infant Gut Microbiota. Microbiol. Mol. Biol. Rev..

[35] LeBlanc J.G., Milani C., de Giori G.S., Sesma F., van Sinderen D., Ventura M. (2013). Bacteria as vitamin suppliers to their host: A gut microbiota perspective. Curr. Opin. Biotechnol..

[36] Wang W., Chen L., Zhou R., Wang X., Song L., Huang S., Wang G., Xia B. (2014). Increased proportions of Bifidobacterium and the Lactobacillus group and loss of butyrate-producing bacteria in inflammatory bowel disease. J. Clin. Microbiol..

[37] Kumari R., Ahuja V., Paul J. (2013). Fluctuations in butyrate-producing bacteria in ulcerative colitis patients of North India. World J. Gastroenterol..

[38] Elinav E., Strowig T., Kau A.L., Henao-Mejia J., Thaiss C.A., Booth C.J., Peaper D.R., Bertin J., Eisenbarth S.C., Gordon J.I. (2011). NLRP6 inflammasome regulates colonic microbial ecology and risk for colitis. Cell.

[39] Ho S., Pothoulakis C., Koon H.W. (2013). Antimicrobial peptides and colitis. Curr. Pharm. Des..

[40] Yan Q., Jia S., Li D., Yang J. (2023). The role and mechanism of action of microbiota-derived short-chain fatty acids in neutrophils: From the activation to becoming potential biomarkers. Biomed. Pharmacother..

[41] Singh N., Thangaraju M., Prasad P.D., Martin P.M., Lambert N.A., Boettger T., Offermanns S., Ganapathy V. (2010). Blockade of dendritic cell development by bacterial fermentation products butyrate and propionate through a transporter (Slc5a8)-dependent inhibition of histone deacetylases. J. Biol. Chem..

[42] Gurav A., Sivaprakasam S., Bhutia Y.D., Boettger T., Singh N., Ganapathy V. (2015). Slc5a8, a Na+-coupled high-affinity transporter for short-chain fatty acids, is a conditional tumour suppressor in colon that protects against colitis and colon cancer under low-fibre dietary conditions. Biochem. J..

[43] Chang P.V., Hao L., Offermanns S., Medzhitov R. (2014). The microbial metabolite butyrate regulates intestinal macrophage function via histone deacetylase inhibition. Proc. Natl. Acad. Sci. USA.

[44] Lin M.Y., de Zoete M.R., van Putten J.P., Strijbis K. (2015). Redirection of Epithelial Immune Responses by Short-Chain Fatty Acids through Inhibition of Histone Deacetylases. Front. Immunol..

[45] Yin L., Laevsky G., Giardina C. (2001). Butyrate suppression of colonocyte NF-kappa B activation and cellular proteasome activity. J. Biol. Chem..

[46] Place R.F., Noonan E.J., Giardina C. (2005). HDAC inhibition prevents NF-kappa B activation by suppressing proteasome activity: Down-regulation of proteasome subunit expression stabilizes I kappa B alpha. Biochem. Pharmacol..

[47] Maul J., Loddenkemper C., Mundt P., Berg E., Giese T., Stallmach A., Zeitz M., Duchmann R. (2005). Peripheral and intestinal regulatory CD4+ CD25(high) T cells in inflammatory bowel disease. Gastroenterology.

[48] Park J., Kim M., Kang S.G., Jannasch A.H., Cooper B., Patterson J., Kim C.H. (2015). Short-chain fatty acids induce both effector and regulatory T cells by suppression of histone deacetylases and regulation of the mTOR-S6K pathway. Mucosal Immunol..

[49] Harrison O.J., Srinivasan N., Pott J., Schiering C., Krausgruber T., Ilott N.E., Maloy K.J. (2015). Epithelial-derived IL-18 regulates Th17 cell differentiation and Foxp3⁺ Treg cell function in the intestine. Mucosal Immunol..

[50] Furusawa Y., Obata Y., Fukuda S., Endo T.A., Nakato G., Takahashi D., Nakanishi Y., Uetake C., Kato K., Kato T. (2013). Commensal microbe-derived butyrate induces the differentiation of colonic regulatory T cells. Nature.

[51] Ricote M., Li A.C., Willson T.M., Kelly C.J., Glass C.K. (1998). The peroxisome proliferator-activated receptor-gamma is a negative regulator of macrophage activation. Nature.

[52] Zhang X., Liu S., Wang Y., Hu H., Li L., Wu Y., Cao D., Cai Y., Zhang J., Zhang X. (2019). Interleukin-22 regulates the homeostasis of the intestinal epithelium during inflammation. Int. J. Mol. Med..

[53] Yang W., Yu T., Huang X., Bilotta A.J., Xu L., Lu Y., Sun J., Pan F., Zhou J., Zhang W. (2020). Intestinal microbiota-derived short-chain fatty acids regulation of immune cell IL-22 production and gut immunity. Nat. Commun..

[54] Vinolo M.A., Rodrigues H.G., Nachbar R.T., Curi R. (2011). Regulation of inflammation by short chain fatty acids. Nutrients.

[55] Morris O., Jasper H. (2021). Reactive Oxygen Species in intestinal stem cell metabolism, fate and function. Free Radic. Biol. Med..

[56] Huang C., Deng W., Xu H.Z., Zhou C., Zhang F., Chen J., Bao Q., Zhou X., Liu M., Li J. (2023). Short-chain fatty acids reprogram metabolic profiles with the induction of reactive oxygen species production in human colorectal adenocarcinoma cells. Comput. Struct. Biotechnol. J..

[57] Tang Y., Chen Y., Jiang H., Nie D. (2011). The role of short-chain fatty acids in orchestrating two types of programmed cell death in colon cancer. Autophagy.

[58] Maslowski K.M., Vieira A.T., Ng A., Kranich J., Sierro F., Yu D., Schilter H.C., Rolph M.S., Mackay F., Artis D. (2009). Regulation of inflammatory responses by gut microbiota and chemoattractant receptor GPR43. Nature.

[59] Tan J., McKenzie C., Potamitis M., Thorburn A.N., Mackay C.R., Macia L. (2014). The role of short-chain fatty acids in health and disease. Adv. Immunol..

[60] Tan Y., Jin Y., Wang Q., Huang J., Wu X., Ren Z. (2019). Perilipin 5 Protects against Cellular Oxidative Stress by Enhancing Mitochondrial Function in HepG2 Cells. Cells.

[61] Oelkrug R., Goetze N., Meyer C.W., Jastroch M. (2014). Antioxidant properties of UCP1 are evolutionarily conserved in mammals and buffer mitochondrial reactive oxygen species. Free Radic. Biol. Med..

[62] Fu X.H., Chen C.Z., Wang Y., Peng Y.X., Wang W.H., Yuan B., Gao Y., Jiang H., Zhang J.B. (2019). COL1A1 affects apoptosis by regulating oxidative stress and autophagy in bovine cumulus cells. Theriogenology.

[63] He Q., Gu L., Lin Q., Ma Y., Liu C., Pei X., Li P.A., Yang Y. (2020). The Immp2l Mutation Causes Ovarian Aging Through ROS-Wnt/β-Catenin-Estrogen Pathway: Preventive Effect of Melatonin. Endocrinology.

[64] Hoste C., Dumont J.E., Miot F., De Deken X. (2012). The type of DUOX-dependent ROS production is dictated by defined sequences in DUOXA. Exp. Cell Res..

[65] Barros L.L., Farias A.Q., Rezaie A. (2019). Gastrointestinal motility and absorptive disorders in patients with inflammatory bowel diseases: Prevalence, diagnosis and treatment. World J. Gastroenterol..

[66] Bassotti G., Antonelli E., Villanacci V., Nascimbeni R., Dore M.P., Pes G.M., Maconi G. (2020). Abnormal gut motility in inflammatory bowel disease: An update. Tech. Coloproctol..

[67] Mawe G.M. (2015). Colitis-induced neuroplasticity disrupts motility in the inflamed and post-inflamed colon. J. Clin. Investig..

[68] da Silva Watanabe P., Cavichioli A.M., D’Arc de Lima Mendes J., Aktar R., Peiris M., Blackshaw L.A., de Almeida Araújo E.J. (2023). Colonic motility adjustments in acute and chronic DSS-induced colitis. Life Sci..

[69] Soret R., Chevalier J., De Coppet P., Poupeau G., Derkinderen P., Segain J.P., Neunlist M. (2010). Short-chain fatty acids regulate the enteric neurons and control gastrointestinal motility in rats. Gastroenterology.

[70] Cherbut C., Ferrier L., Rozé C., Anini Y., Blottière H., Lecannu G., Galmiche J.P. (1998). Short-chain fatty acids modify colonic motility through nerves and polypeptide YY release in the rat. Am. J. Physiol..

[71] Shaidullov I.F., Sorokina D.M., Sitdikov F.G., Hermann A., Abdulkhakov S.R., Sitdikova G.F. (2021). Short chain fatty acids and colon motility in a mouse model of irritable bowel syndrome. BMC Gastroenterol..

[72] Wang X., Liang F., Dai Z., Feng X., Qiu F. (2024). Combination of Coptis chinensis polysaccharides and berberine ameliorates ulcerative colitis by regulating gut microbiota and activating AhR/IL-22 pathway. J. Ethnopharmacol..

[73] Sun X., Zhang Y., Cheng G., Zhu T., Zhang Z., Xiong L., Hu H., Liu H. (2023). Berberine improves DSS-induced colitis in mice by modulating the fecal-bacteria-related bile acid metabolism. Biomed. Pharmacother..

[74] Liu C.S., Liang X., Wei X.H., Jin Z., Chen F.L., Tang Q.F., Tan X.M. (2019). Gegen Qinlian Decoction Treats Diarrhea in Piglets by Modulating Gut Microbiota and Short-Chain Fatty Acids. Front. Microbiol..

[75] Hänninen A., Toivonen R., Pöysti S., Belzer C., Plovier H., Ouwerkerk J.P., Emani R., Cani P.D., De Vos W.M. (2018). Akkermansia muciniphila induces gut microbiota remodelling and controls islet autoimmunity in NOD mice. Gut.

[76] Xu J., Lian F., Zhao L., Zhao Y., Chen X., Zhang X., Guo Y., Zhang C., Zhou Q., Xue Z. (2015). Structural modulation of gut microbiota during alleviation of type 2 diabetes with a Chinese herbal formula. ISME J..

[77] Wang X., Quan J., Xiu C., Wang J., Zhang J. (2023). Gegen Qinlian decoction (GQD) inhibits ulcerative colitis by modulating ferroptosis-dependent pathway in mice and organoids. Chin. Med..

[78] Wang Z., Shu W., Zhao R., Liu Y., Wang H. (2023). Sodium butyrate induces ferroptosis in endometrial cancer cells via the RBM3/SLC7A11 axis. Apoptosis Int. J. Program. Cell Death.

[79] Bian Z., Sun X., Liu L., Qin Y., Zhang Q., Liu H., Mao L., Sun S. (2023). Sodium Butyrate Induces CRC Cell Ferroptosis via the CD44/SLC7A11 Pathway and Exhibits a Synergistic Therapeutic Effect with Erastin. Cancers.

[80] Li R., Chen Y., Shi M., Xu X., Zhao Y., Wu X., Zhang Y. (2016). Gegen Qinlian decoction alleviates experimental colitis via suppressing TLR4/NF-κB signaling and enhancing antioxidant effect. Phytomedicine.

[81] Hu J., Huang H., Che Y., Ding C., Zhang L., Wang Y., Hao H., Shen H., Cao L. (2021). Qingchang Huashi Formula attenuates DSS-induced colitis in mice by restoring gut microbiota-metabolism homeostasis and goblet cell function. J. Ethnopharmacol..

[82] Yuan X., Wang L., Bhat O.M., Lohner H., Li P.L. (2018). Differential effects of short chain fatty acids on endothelial Nlrp3 inflammasome activation and neointima formation: Antioxidant action of butyrate. Redox Biol..

[83] Niu C., Hu X.L., Yuan Z.W., Xiao Y., Ji P., Wei Y.M., Hua Y.L. (2023). Pulsatilla decoction improves DSS-induced colitis via modulation of fecal-bacteria-related short-chain fatty acids and intestinal barrier integrity. J. Ethnopharmacol..

[84] Wang X., Xu L., Wang T., Xu J., Fan F., Zhang Y., Wang J., Cao Q. (2022). Pulsatilla decoction alleviates colitis by enhancing autophagy and regulating PI3K-Akt-mTORC1 signaling pathway. Mol. Med. Rep..

[85] Li Z., Song Y., Xu W., Chen J., Zhou R., Yang M., Zhu G., Luo X., Ai Z., Liu Y. (2023). Pulsatilla chinensis saponins improve SCFAs regulating GPR43-NLRP3 signaling pathway in the treatment of ulcerative colitis. J. Ethnopharmacol..

[86] Tang S., Liu W., Zhao Q., Li K., Zhu J., Yao W., Gao X. (2021). Combination of polysaccharides from Astragalus membranaceus and Codonopsis pilosula ameliorated mice colitis and underlying mechanisms. J. Ethnopharmacol..

[87] Shao S., Wang D., Zheng W., Li X., Zhang H., Zhao D., Wang M. (2019). A unique polysaccharide from Hericium erinaceus mycelium ameliorates acetic acid-induced ulcerative colitis rats by modulating the composition of the gut microbiota, short chain fatty acids levels and GPR41/43 respectors. Int. Immunopharmacol..

[88] Ren Y., Geng Y., Du Y., Li W., Lu Z.M., Xu H.Y., Xu G.H., Shi J.S., Xu Z.H. (2018). Polysaccharide of Hericium erinaceus attenuates colitis in C57BL/6 mice via regulation of oxidative stress, inflammation-related signaling pathways and modulating the composition of the gut microbiota. J. Nutr. Biochem..

[89] Yu Z., Li D., Sun H. (2023). Herba Origani alleviated DSS-induced ulcerative colitis in mice through remolding gut microbiota to regulate bile acid and short-chain fatty acid metabolisms. Biomed. Pharmacother..

[90] Zheng J., Li H., Zhang P., Yue S., Zhai B., Zou J., Cheng J., Zhao C., Guo D., Wang J. (2022). Paeonol Ameliorates Ulcerative Colitis in Mice by Modulating the Gut Microbiota and Metabolites. Metabolites.

[91] Li M.Y., Luo H.J., Wu X., Liu Y.H., Gan Y.X., Xu N., Zhang Y.M., Zhang S.H., Zhou C.L., Su Z.R. (2019). Anti-Inflammatory Effects of Huangqin Decoction on Dextran Sulfate Sodium-Induced Ulcerative Colitis in Mice Through Regulation of the Gut Microbiota and Suppression of the Ras-PI3K-Akt-HIF-1α and NF-κB Pathways. Front. Pharmacol..

[92] Zhu J.J., Liu H.Y., Yang L.J., Fang Z., Fu R., Chen J.B., Liu S., Fei B.Y. (2023). Anti-tumour effect of Huangqin Decoction on colorectal cancer mice through microbial butyrate mediated PI3K/Akt pathway suppression. J. Med. Microbiol..

[93] Wang Y., Zhu X., Liang Y., Li X., Wang Y., Li J. (2022). Sishen Wan Treats Ulcerative Colitis in Rats by Regulating Gut Microbiota and Restoring the Treg/Th17 Balance. Evid. Based Complement. Altern. Med..

[94] Chen M.J., Feng Y., Gao L., Lin M.X., Wang S.D., Tong Z.Q. (2023). Composite Sophora Colon-Soluble Capsule Ameliorates DSS-Induced Ulcerative Colitis in Mice via Gut Microbiota-Derived Butyric Acid and NCR^+^ ILC3. Chin. J. Integr. Med..

[95] Fernández J., de la Fuente V.G., García M.T.F., Sánchez J.G., Redondo B.I., Villar C.J., Lombó F. (2020). A diet based on cured acorn-fed ham with oleic acid content promotes anti-inflammatory gut microbiota and prevents ulcerative colitis in an animal model. Lipids Health Dis..

[96] Li J., Ma Y., Li X., Wang Y., Huo Z., Lin Y., Li J., Yang H., Zhang Z., Yang P. (2022). Fermented Astragalus and its metabolites regulate inflammatory status and gut microbiota to repair intestinal barrier damage in dextran sulfate sodium-induced ulcerative colitis. Front. Nutr..

[97] Sun Z., Li J., Dai Y., Wang W., Shi R., Wang Z., Ding P., Lu Q., Jiang H., Pei W. (2020). Indigo Naturalis Alleviates Dextran Sulfate Sodium-Induced Colitis in Rats via Altering Gut Microbiota. Front. Microbiol..

[98] Su L., Mao C., Wang X., Li L., Tong H., Mao J., Ji D., Lu T., Hao M., Huang Z. (2020). The Anti-colitis Effect of Schisandra chinensis Polysaccharide Is Associated With the Regulation of the Composition and Metabolism of Gut Microbiota. Front. Cell. Infect. Microbiol..

[99] Xuan H., Ou A., Hao S., Shi J., Jin X. (2020). Galangin Protects against Symptoms of Dextran Sodium Sulfate-induced Acute Colitis by Activating Autophagy and Modulating the Gut Microbiota. Nutrients.

[100] Hu L., Wu C., Zhang Z., Liu M., Maruthi Prasad E., Chen Y., Wang K. (2019). Pinocembrin Protects Against Dextran Sulfate Sodium-Induced Rats Colitis by Ameliorating Inflammation, Improving Barrier Function and Modulating Gut Microbiota. Front. Physiol..

[101] Zimmerman M.A., Singh N., Martin P.M., Thangaraju M., Ganapathy V., Waller J.L., Shi H., Robertson K.D., Munn D.H., Liu K. (2012). Butyrate suppresses colonic inflammation through HDAC1-dependent Fas upregulation and Fas-mediated apoptosis of T cells. Am. J. Physiol. Gastrointest. Liver Physiol..

[102] Segain J.P., Raingeard de la Blétière D., Bourreille A., Leray V., Gervois N., Rosales C., Ferrier L., Bonnet C., Blottière H.M., Galmiche J.P. (2000). Butyrate inhibits inflammatory responses through NFkappaB inhibition: Implications for Crohn’s disease. Gut.

[103] Liu T., Li J., Liu Y., Xiao N., Suo H., Xie K., Yang C., Wu C. (2012). Short-chain fatty acids suppress lipopolysaccharide-induced production of nitric oxide and proinflammatory cytokines through inhibition of NF-κB pathway in RAW264.7 cells. Inflammation.

[104] Lee C., Kim B.G., Kim J.H., Chun J., Im J.P., Kim J.S. (2017). Sodium butyrate inhibits the NF-kappa B signaling pathway and histone deacetylation, and attenuates experimental colitis in an IL-10 independent manner. Int. Immunopharmacol..

[105] Zhang C.L., Zhang S., He W.X., Lu J.L., Xu Y.J., Yang J.Y., Liu D. (2017). Baicalin may alleviate inflammatory infiltration in dextran sodium sulfate-induced chronic ulcerative colitis via inhibiting IL-33 expression. Life Sci..

[106] Yin M., Zhang Y., Li H. (2019). Advances in Research on Immunoregulation of Macrophages by Plant Polysaccharides. Front. Immunol..

[107] Fu Y.P., Feng B., Zhu Z.K., Feng X., Chen S.F., Li L.X., Yin Z.Q., Huang C., Chen X.F., Zhang B.Z. (2018). The Polysaccharides from Codonopsis pilosula Modulates the Immunity and Intestinal Microbiota of Cyclophosphamide-Treated Immunosuppressed Mice. Molecules.

[108] Liu Y.J., Tang B., Wang F.C., Tang L., Lei Y.Y., Luo Y., Huang S.J., Yang M., Wu L.Y., Wang W. (2020). Parthenolide ameliorates colon inflammation through regulating Treg/Th17 balance in a gut microbiota-dependent manner. Theranostics.

[109] Sun M., Wu W., Liu Z., Cong Y. (2017). Microbiota metabolite short chain fatty acids, GPCR, and inflammatory bowel diseases. J. Gastroenterol..

[110] Dalile B., Van Oudenhove L., Vervliet B., Verbeke K. (2019). The role of short-chain fatty acids in microbiota-gut-brain communication. Nat. Rev. Gastroenterol. Hepatol..

[111] Eastaff-Leung N., Mabarrack N., Barbour A., Cummins A., Barry S. (2010). Foxp3+ regulatory T cells, Th17 effector cells, and cytokine environment in inflammatory bowel disease. J. Clin. Immunol..

[112] Britton G.J., Contijoch E.J., Mogno I., Vennaro O.H., Llewellyn S.R., Ng R., Li Z., Mortha A., Merad M., Das A. (2019). Microbiotas from Humans with Inflammatory Bowel Disease Alter the Balance of Gut Th17 and RORγt(+) Regulatory T Cells and Exacerbate Colitis in Mice. Immunity.

[113] Xu X., Wang Y., Wei Z., Wei W., Zhao P., Tong B., Xia Y., Dai Y. (2017). Madecassic acid, the contributor to the anti-colitis effect of madecassoside, enhances the shift of Th17 toward Treg cells via the PPARγ/AMPK/ACC1 pathway. Cell Death Dis..

[114] Gálvez J. (2014). Role of Th17 Cells in the Pathogenesis of Human IBD. ISRN Inflamm..

[115] Venkataraman B., Ojha S., Belur P.D., Bhongade B., Raj V., Collin P.D., Adrian T.E., Subramanya S.B. (2020). Phytochemical drug candidates for the modulation of peroxisome proliferator-activated receptor γ in inflammatory bowel diseases. Phytother. Res..

[116] Peng L., Li Z.R., Green R.S., Holzman I.R., Lin J. (2009). Butyrate enhances the intestinal barrier by facilitating tight junction assembly via activation of AMP-activated protein kinase in Caco-2 cell monolayers. J. Nutr..

[117] Macia L., Tan J., Vieira A.T., Leach K., Stanley D., Luong S., Maruya M., Ian McKenzie C., Hijikata A., Wong C. (2015). Metabolite-sensing receptors GPR43 and GPR109A facilitate dietary fibre-induced gut homeostasis through regulation of the inflammasome. Nat. Commun..

[118] Tourkochristou E., Aggeletopoulou I., Konstantakis C., Triantos C. (2019). Role of NLRP3 inflammasome in inflammatory bowel diseases. World J. Gastroenterol..

[119] Kim M.H., Kang S.G., Park J.H., Yanagisawa M., Kim C.H. (2013). Short-chain fatty acids activate GPR41 and GPR43 on intestinal epithelial cells to promote inflammatory responses in mice. Gastroenterology.

[120] Bagalagel A., Diri R., Noor A., Almasri D., Bakhsh H.T., Kutbi H.I., Al-Gayyar M.M.H. (2022). Curative effects of fucoidan on acetic acid induced ulcerative colitis in rats via modulating aryl hydrocarbon receptor and phosphodiesterase-4. BMC Complement. Med. Ther..

[121] Qiu J., Zhou L. (2013). Aryl hydrocarbon receptor promotes RORγt⁺ group 3 ILCs and controls intestinal immunity and inflammation. Semin. Immunopathol..

[122] Hou J.J., Ma A.H., Qin Y.H. (2023). Activation of the aryl hydrocarbon receptor in inflammatory bowel disease: Insights from gut microbiota. Front. Cell. Infect. Microbiol..

[123] Kawai S., Iijima H., Shinzaki S., Hiyama S., Yamaguchi T., Araki M., Iwatani S., Shiraishi E., Mukai A., Inoue T. (2017). Indigo Naturalis ameliorates murine dextran sodium sulfate-induced colitis via aryl hydrocarbon receptor activation. J. Gastroenterol..

[124] Smith P.M., Howitt M.R., Panikov N., Michaud M., Gallini C.A., Bohlooly Y.M., Glickman J.N., Garrett W.S. (2013). The microbial metabolites, short-chain fatty acids, regulate colonic Treg cell homeostasis. Science.

[125] Wang M., Fu R., Xu D., Chen Y., Yue S., Zhang S., Tang Y. (2024). Traditional Chinese Medicine: A promising strategy to regulate the imbalance of bacterial flora, impaired intestinal barrier and immune function attributed to ulcerative colitis through intestinal microecology. J. Ethnopharmacol..

